# Vapor Pressure of Selected Aliphatic Hexanols by Static and Indirect Chromatographic Methods

**DOI:** 10.3390/molecules30214287

**Published:** 2025-11-04

**Authors:** Vojtěch Štejfa, Pavel Šimáček, Bohumír Koutek, Michal Fulem, Květoslav Růžička

**Affiliations:** 1 Department of Physical Chemistry, University of Chemistry and Technology, CZ-166 28 Prague, Czech Republic; bkoutek@seznam.cz (B.K.); fulemm@vscht.cz (M.F.); 2 Department of Sustainable Fuels and Green Chemistry, University of Chemistry and Technology, CZ-166 28 Prague, Czech Republic; simacekp@vscht.cz

**Keywords:** vapor pressures, aliphatic hexanols, static method, indirect chromatographic method, ideal gas properties

## Abstract

Vapor pressures of eight aliphatic hexanols ((±)-3-hexanol, CAS RN: 623-37-0; 2-methyl-2-pentanol, CAS RN: 590-36-3; (±)-2-methyl-3-pentanol, CAS RN: 565-67-3; (±)-3-methyl-2-pentanol, CAS RN: 565-60-6; 3-methyl-3-pentanol, CAS RN: 77-74-7; 2,2-dimethyl-1-butanol, CAS RN: 1185-33-7; 2,3-dimethyl-2-butanol, CAS RN: 594-60-5; and (±)-3,3-dimethyl-2-butanol, CAS RN: 464-07-3) were measured by the static method in the temperature range of 233 to 308 K. These data were combined with selected literature vapor pressures and simultaneously correlated with heat capacities in the ideal gaseous state (determined in the framework of this work, since no literature data were available) and liquid heat capacities reported by us previously. The vapor pressures measured for test (*p*_x_) and reference (*p*_r_) compounds were combined with corresponding gas–liquid chromatographic (GLC) adjusted retention times (*t*′) measured in the same temperature region to determine relative activity coefficients at infinite dilution ($\gamma_{\mathrm{rel}}^{\infty }$). The linearly extrapolated values of $\gamma_{\mathrm{rel}}^{\infty }$ up to 363 K, together with known directly measured *p*_r_ values at these temperatures, allow reasonably accurate *p*_x_ data to be obtained at extrapolated temperatures. Results were compared with fragmentary literature data. Enthalpies of vaporization derived from the vapor pressures obtained in this work represent a significant contribution to existing databases.

## 1. Introduction

Aliphatic monohydroxy alcohols are an important class of organic compounds, widely distributed in nature and extensively used in commercial and industrial applications—for example, as solvents, intermediates in plasticizer production [1,2], and even as alternative fuels [3,4,5]. Although structurally similar to aliphatic alkanes, alcohols exhibit markedly different physical properties due to their high dipole moments and their ability to form hydrogen bonds (H-bonds) [6,7,8].

Hydrogen bonding has been the subject of numerous studies, employing a wide spectrum of experimental and computational techniques, with the primary focus on understanding this phenomenon at the molecular level [9,10,11,12]. However, the propagation of H-bond effects to macroscopic properties—such as liquid densities, *pVT* behavior in the gas phase, liquid heat capacities, and vapor pressures—has received comparatively less attention. Most of the available data concern alcohols with fewer than five carbon atoms, as documented in the comprehensive handbook by Wilhoit and Zwolinski [13], which summarizes data published up to 1968. Although the volume of experimental data has grown substantially since then, the majority of investigations still focus on primary alkanols (even for higher homologs [14,15,16]) and secondary alcohols [17,18,19,20], leaving many branched isomers insufficiently characterized. This gap hinders a deeper understanding of the relationship between molecular structure, hydrogen-bond strength, and the nature of associated oligomers, as well as the development of accurate estimation methods required for engineering applications.

In previous studies, including those focused on group-contribution methods, aliphatic alcohols have typically been classified as primary, secondary, or tertiary based on the nature of the carbon atom (C_α_) directly bonded to the hydroxyl group. Primary alcohols have one carbon atom (C_β_) bonded to C_α_ (*N*_β_ = 1), secondary alcohols have two (*N*_β_ = 2), and tertiary alcohols have three (*N*_β_ = 3). This classification is usually associated with steric hindrance, with the assumption that increasing *N*_β_ weakens hydrogen bonding. Our previous work dealing with liquid heat capacities of all 17 constitutional hexanol isomers [21], however, indicated that not only the number of β-carbons but also the number of γ-carbons (*N*_γ_) plays a significant role. Thus, the combined parameter *N*_β+γ_ serves as a better descriptor of steric effects on hydrogen-bond strength.

Due to their molecular variability regarding length, branching of the alkyl chain, and position of the hydroxyl group, aliphatic hexanols (C_6_H_14_O) are considered an appropriate system for studying/understanding their physicochemical properties and/or supramolecular organization. Assuming their vapor pressures might be even more sensitive than liquid heat capacities for quantifying structure–property relationships, we decided to determine the vapor pressures of a subset of eight hexanols, despite the fact that the vapor pressures of many of these compounds have been measured by different techniques in various laboratories. The interlaboratory agreement of the literature data is, however, poor, preventing the derivation of reliable conclusions. Thus, the present study focuses on those hexanols that belong to the group with the highest *N*_β+γ_ values (4 or 5) among all constitutional hexanol isomers. Namely, the following hexanols were studied: one primary alcohol (2,2-dimethyl-1-butanol, hereinafter referred to as 22M1B, *N*_β+γ_ = 4); four secondary alcohols ((±)-3-hexanol (3H), *N*_β+γ_ = 4; (±)-2-methyl-3-pentanol (2M3P), *N*_β+γ_ = 5; (±)-3-methyl-2-pentanol (3M2P), *N*_β+γ_ = 4; and (±)-3,3-dimethyl-2-butanol (33M2B), *N*_β+γ_ = 5); and three tertiary alcohols (2-methyl-2-pentanol (2M2P), *N*_β+γ_ = 4; 3-methyl-3-pentanol (3M3P), *N*_β+γ_ = 5; and 2,3-dimethyl-2-butanol (23M2B), *N*_β+γ_ = 5). Among these, only three (2M2P, 3M3P, and 22M1B) are produced and used in significant quantities. Our selection, however, was guided not by industrial relevance but by structural factors expected to influence hydrogen bonding.

By determining the vapor pressures of these selected aliphatic hexanols in the temperature range of approximately 233–308 K using the static method, we aim to provide further insight into the interplay between molecular structure, steric hindrance, and hydrogen-bond strength in this important class of compounds. An additional outcome of this work is the determination of vaporization enthalpies, including values at the most frequently required temperature of 298.15 K. For several compounds (3M3P, 22M1B, 23M1B, and 33M2B) we were also able to determine sublimation pressures.

The data obtained in this work can also be compared with those for 1-hexanol, which exhibits stronger H-bonding and for which we recently developed recommended vapor-pressure values covering the range from 238 K to *T*_nbp_ [22].

Ideal-gas heat capacities were also determined as part of this study. This allowed us to verify the thermodynamic consistency between the vapor pressures obtained here, the literature data, and the heat capacities of both the liquid and gaseous phases.

The resulting vapor-pressure data were further used to evaluate the potential of extending reliable measurements—available only within a limited temperature range—toward higher temperatures using a relatively fast indirect gas–liquid chromatographic method. There is a relation between chromatographic isothermal retention times and vapor pressures; however, our previous studies have shown that using this indirect chromatographic method alone introduces systematic bias [23,24]. Nonetheless, its combination with a direct vapor-pressure measurement could yield data of acceptable accuracy.

## 2. Results

This section describes the experimental values obtained within this framework, i.e., vapor pressures *p* (Section 2.1, *U*_c_ (*p*/Pa) = 0.005 (*p*/Pa) + 0.05, see Section 4.2), gas–liquid chromatographic retention times (Section 2.2, *u*_r_ = 0.005, see Section 4.5), and heat capacities in the state of ideal gas (Section 2.3, *u*_r_ = 0.006, see Table S5 in the Supplementary Materials).

### 2.1. Vapor Pressures by the Static Method

Experimental vapor pressures were determined using the static method with a capacitance diaphragm gauge (CDG), internally designated as STAT6, which is capable of measuring pressures in the range of approximately 0.5–1333 Pa (for details, see Section 4.2). Measurements were performed over a temperature range of approximately 233–308 K, the upper limit being defined by the operating range of the STAT6 gauge. For some compounds, measurements began at temperatures slightly above 233 K due to extremely low vapor pressures at the lower limit of the range.

**Figure 1:**
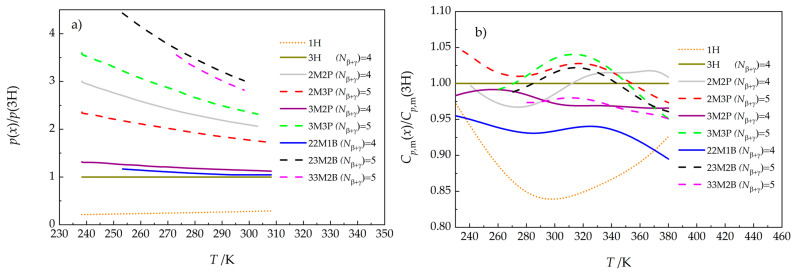
Ratio of (**a**) liquid vapor pressures *p*(*x*) and (**b**) liquid heat capacities *C_p_*_,m_(*x*) [21] of hexanols studied in this work relative to 3-hexanol (3H). Data for 1-hexanol [21,22] are shown for comparison (see Table 5 for compound abbreviations).

For 3M3P (*T*_crI-l_ = 250.4 K [21]), 22M1B (*T*_crI-l_ = 251.2 K [21]), 23M2B (*T*_crI-l_ = 261.6 K [21]), and 33M2B (*T*_crI-l_ = 278.4 K [21]), sublimation pressures were also determined for the Crystal I phase. In the case of 33M2B (*T*_crII-crI_ = 250.0 K [21]), additional data for the Crystal II phase were also obtained, and the liquid phase was studied in the short temperature range of 273–298 K. For 23M2B and 33M2B, the vapor pressures for the supercooled liquid were determined within a limited temperature range. The complete set of measured data is provided in Table S2 in the Supplementary Materials. To highlight the difference between the respective hexanols, we present the ratio of vapor pressures to those of the least volatile compound, 3H (see Figure 1). Note, however, that the 3H vapor pressure was still about five times higher than that of 1-hexanol at 233 K and about three times higher at 308 K. The most volatile compound was 23M2B, exhibiting vapor pressures at least three times higher than those of 3H across the studied range.

It should be noted that differences in liquid heat capacities [21] between the same set of compounds were less than 10 percent (and less than 17 percent for 1-hexanol; see Figure 1b).

### 2.2. Gas–Liquid Chromatographic Retention Times

Experimental gas–liquid chromatographic isothermal retention times (GLC-RT) were determined using a commercial chromatograph, as described in Section 4.4. This setup allowed GLC-RT measurements to be carried out in the temperature range of 283–363 K, providing an overlap with the range covered by the static method. The measured data are presented in Table 1.

Retention times listed in Table 1 are related to vapor pressures via Equation (1), derived in 1957 by Herington [25]. In principle, it is based on the concept that the retention times of single compounds are inversely correlated with their respective vapor pressures:

(1) $$\frac{t_{x}^{\prime }(T)}{t_{r}^{\prime }(T)}=\frac{\gamma_{r}^{\infty }(T)\cdot p_{r}(T)}{\gamma_{x}^{\infty }(T)\cdot p_{x}(T)}=\gamma_{\mathrm{rel}}^{\infty }(T)\frac{p_{r}(T)}{p_{x}(T)}$$

where *t*′ = *t_i_* − *t*_0_ is the adjusted retention time (*t_i_* and *t*_0_ are the retention times of the solute and unretained solute, respectively), *p_i_* is the saturated vapor pressure, and $\gamma_{i}^{\infty }$ is the limiting activity coefficient of solute *i* in the stationary phase (*i* = x for the test compound and *i* = r for the reference compound). This relation enables vapor-pressure determination, provided the data for the reference compound *p*_r_ are known. The procedure is described in Section 4.6, and the results obtained in this way are discussed in Section 3.5.

### 2.3. Heat Capacities in the Ideal-Gas State

Geometries, relative conformer energies, point-group symmetries, and products of the principal moments of inertia of all conformers used in the R1TM approach [22] are listed in Table S3 in the Supplementary Materials, and the parameters of internal rotations (internal symmetry number, reduced moment of inertia, and Fourier expansion parameters) are listed in Table S4 in the Supplementary Materials. The thermodynamic properties calculated using the R1TM approach are presented in Table S5 in the Supplementary Materials. Entropies of the chiral compounds are reported for racemic mixtures (*R*ln2 higher than for enantiopure compounds), except for 3M2P. 3M2P has four stereoisomers and the ideal-gas properties of (2*R*,3*R*)-isomer (equal to (2*S*,3*S*)) and (2*R*,3*S*)-isomer (equal to (2S,3*R*)) are reported separately. Since the $C_{p,m}^{g0}$ values of 3M2P isomers differ by less than 1 J K^−1^ mol^−1^, their arithmetic average was used in the SimCor procedure (Section 3.2).

No data were found for comparison, but it should be noted that differences in $C_{p,m}^{g0}$ among the eight compounds studied (even when extended by 1-hexanol [22]) are very small. At all temperatures over the range 200 to 700 K, the largest difference between any two isomers is below *R*. This contrasts sharply with the heat capacities of the liquid hexanols, which differ noticeably among the isomers. For example, the difference between the heat capacities of 3M3P and 1-hexanol was observed to reach 60 J K^−1^ mol^−1^ at 310 K [21] (see also Figure 1b).

## 3. Discussion

This section contains a critical comparison of the data obtained in this work with literature values and correlation of selected data sources using the Cox eq., Equation (3) (Section 4.4). A comparison of derived evaporation enthalpies with previously published values and of vapor pressures obtained by combining static and indirect chromatographic methods with reliable data from the literature is also provided.

### 3.1. Vapor Pressures

A summary of the vapor-pressure experiments performed in this work is presented in Table 2, along with the available literature data.

In the evaluation of vapor pressures, we start with three compounds for which ebulliometric data up to the normal boiling-point temperature are available from Belarus State University (BSU): 3H [18], 2M2P [26], and 2M3P [17]. Data from this laboratory for other alcohols generally agree reasonably well with results from other groups. For example, 2-octanol data are consistent with those obtained by the ebulliometric method by Ambrose and Ghiassee [27] (except for some points below 10 kPa), while 2,2-dimethyl-1-propanol and several other alcohols (2-methyl-1-butanol, 2-methyl-2-butanol, 3-methyl-2-butanol, 2-pentanol, and 3-pentanol) match data from two distinct ebulliometric apparatuses published by Čenský et al. [28,29]. This agreement (within a few hundred pascals at *T*_nbp_) was one of the reasons why we also used BSU data in our previous work [24].

**Figure 2:**
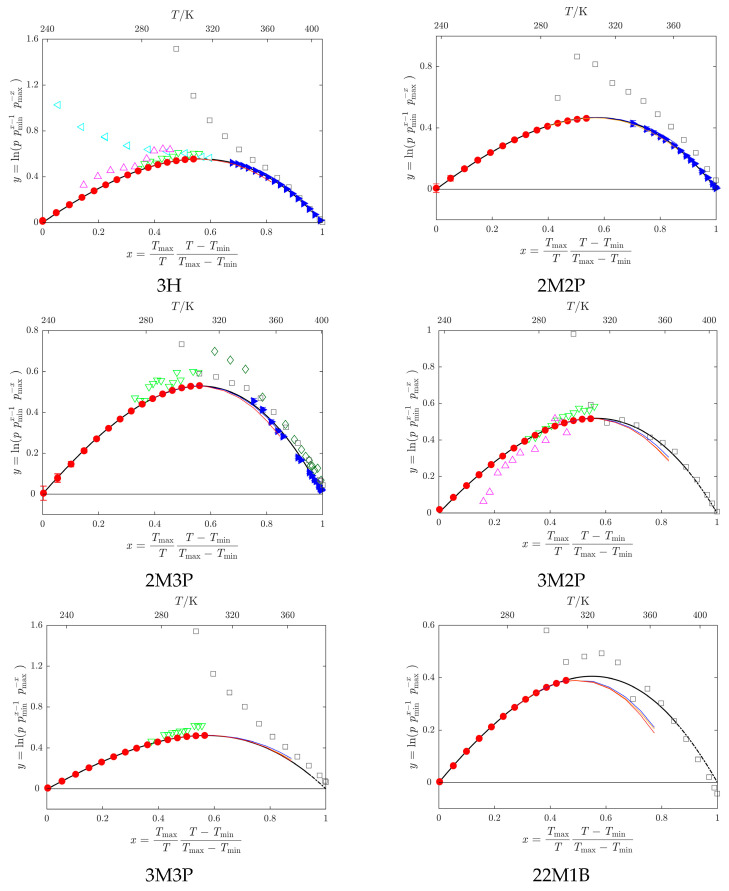

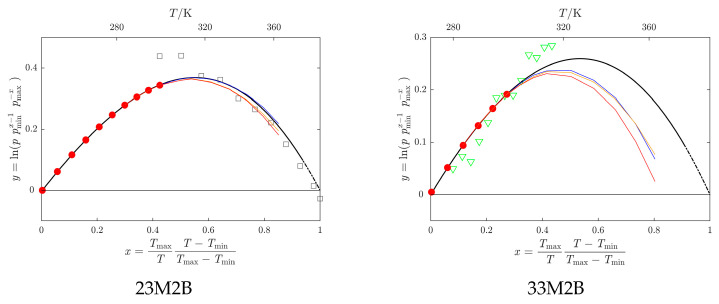
Arc-plot representation [28] of vapor pressures (see Table 5 for compound abbreviations). 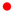 This work; 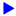 Belarus State University—BSU (3H: Sachek et al. [18]; 2M2P: Markovnik et al. [26]; 2M3P: Brazhnikov et al. [17]); 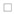 Hovorka et al. (3H: [33], 2M2P: [34], 2M3P and 3M3P [35], 3M2P and 23M2B [36], 22M1B [38]); 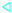 N’Guimbi et al. [20]; 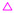 Thomas et al. [31]; 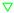 Kulikov et al. [32]; 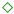 Loginova et al. [40]. Vapor pressures obtained by a combination of static and indirect chromatographic methods (GLC-ACRT (Activity Coefficients–Retention Times) method, Section 3.5 and Section 4.6): **−** reference 2M2P, **−** reference 2M3P, **−** reference 3H. 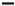, data obtained by the SimCor procedure (Section 3.2 and Section 4.4). Data sets represented by filled symbols were used in the SimCor procedure.

For each compound, the data listed in Table 2 were first converted to ITS-90 according to the procedure reported by Goldberg and Weir [30]. As a first step, literature data and the results of this work for 3H, 2M2P, and 2M3P were compared graphically in Figure 2 using the arc-plot representation [28].

For 3H, the static vapor pressures determined in this work differ from those reported in the literature [20,31,32,33]. Data measured by Hovorka et al. [33] from approximately 1 kPa to 100 kPa using the isoteniscope method are systematically higher than both our values and those obtained by BSU. The same observation applies to 2M2P [34] and 2M3P [35]. Hovorka and co-workers also reported vapor pressures for other hexanols considered in this study [36,37,38] and for 1-hexanol [33], with similar discrepancies. At the time these data were published (the 1930s and 1940s), sample purity could only be assessed indirectly, through properties such as density and refractive index.

Values reported by Thomas et al. [31] are higher than our data for 3H but lower for 3M2P. The Ramsay–Young method was employed in their work, and purity was again specified only indirectly. Furthermore, temperatures were reported to just one decimal place and pressure to two significant digits. Any attempt to explain observed differences would therefore be speculative.

Data for 3H by N’Guimbi et al. [20] were obtained by a static method similar to that used in the present study. Data from the Lyon laboratory typically agree well with our results, as demonstrated for 1-alkanols [39], which are consistent with Ambrose et al. [15] and with our own data [22]. The reason for the large discrepancies in 3H vapor pressures remains unclear.

**Table 2:** Overview of vapor pressures of the studied compounds ^a^.

Reference	Phase	*N* ^b^	(*T*_min_–*T*_max_)/K	(*p*_min_–*p*_max_)/kPa	Method
	3H				
Hovorka et al. [33]	Liq	13	298.15–411.15	0.960–109.391	Isoteniscope ^c^
Thomas et al. [31]	Liq	9	253.95–294.75	0.006–0.306	Ramsay–Young ^c^
**Sachek et al. [18]**	**Liq**	**16**	**333.27** **–** **408.93**	**3.901** **–** **101.661**	**Ebulliometry**
N’Guimbi et al. [20]	Liq	11	243.94–318.15	0.004–1.549	Static
Kulikov et al. [32]	Liq	11	278.3–311.5	0.067–1.020	Transpiration
**This work**	**Liq**	**15**	**238.30** **–** **308.16**	**0.001** **–** **0.768**	**Static**
	2M2P				
Hovorka et al. [34]	Liq	12	288.15–396.15	0.400–107.858	Isoteniscope ^c^
**Markovnik et al. [26]**	**Liq**	**15**	**331.07** **–** **396.39**	**6.37** **–** **103.76**	**Ebulliometry**
**This work**	**Liq**	**14**	**238.30** **–** **303.16**	**0.002** **–** **1.097**	**Static**
	2M3P				
Hovorka et al. [35]	Liq	13	298.15–401.15	0.800–105.325	Isoteniscope ^c^
Loginova et al. [40]	Liq	14	317.24–399.52	2.853–101.112	Ebulliometry
**Brazhnikov et al. [17]**	**Liq**	**15**	**343.07** **–** **400.02**	**10.376** **–** **98.260**	**Ebulliometry**
Kulikov et al. [32]	Liq	13	274.9–307.5	0.098–1.350	Transpiration
**This work**	**Liq**	**16**	**233.29** **–** **308.15**	**0.001** **–** **1.322**	**Static**
	3M2P				
Hovorka et al. [36]	Liq	13	298.15–408.15	0.667–103.325	Isoteniscope ^c^
Thomas et al. [31]	Liq	11	255.05–294.45	0.006–0.293	Ramsay–Young ^c^
Kulikov et al. [32]	Liq	13	275.1–310.3	0.058–1.076	Transpiration
**This work**	**Liq**	**15**	**238.24** **–** **308.09**	**0.001** **–** **0.862**	**Static**
	3M3P				
Hovorka et al. [35]	Liq	12	298.15–394.15	2.400–101.698	Isoteniscope ^c^
Kulikov et al. [32]	Liq	11	275.2–301.5	0.137–1.212	Transpiration
**This work**	**Liq**	**15**	**233.30** **–** **303.11**	**0.001** **–** **1.232**	**Static**
**This work**	**Cr I**	**4**	**233.31** **–** **248.37**	**0.001** **–** **0.008**	**Static**
	22M1B				
Hovorka et al. [38]	Liq	14	298.15–415.95	0.400–121.590	Isoteniscope ^c^
**This work**	**Liq**	**12**	**253.26** **–** **308.15**	**0.005** **–** **0.784**	**Static**
**This work**	**Cr I**	**3**	**238.80** **–** **248.27**	**0.001** **–** **0.003**	**Static**
	23M2B				
Hovorka et al. [36]	Liq	11	298.15–393.15	1.120–105.191	Isoteniscope ^c^
**This work**	**Liq**	**10**	**253.28** **–** **298.16**	**0.020** **–** **1.093**	**Static**
**This work**	**Cr I**	**5**	**238.30** **–** **258.26**	**0.002** **–** **0.032**	**Static**
	33M2B				
Kulikov et al. [32]	Liq	13	279.9–315.3	0.248–3.254	Transpiration
**This work**	**Liq**	**6**	**273.24** **–** **298.13**	**0.142** **–** **1.021**	**Static**
**This work**	**Cr I**	**7**	**248.33** **–** **278.22**	**0.009** **–** **0.218**	**Static**
**This work**	**Cr II**	**5**	**233.73** **–** **253.32**	**0.001** **–** **0.016**	**Static**

^a^ References containing single vapor pressure point are not listed. Data sets printed in bold were used in the SimCor procedure (Section 3.2 and Section 4.4). See Table 5 for compound abbreviations. ^b^ Number of points. ^c^ Isoteniscope (which is essentially a static method) and Ramsay–Young method are not used anymore; for details, see review by Ambrose [41].

**Figure 3:**
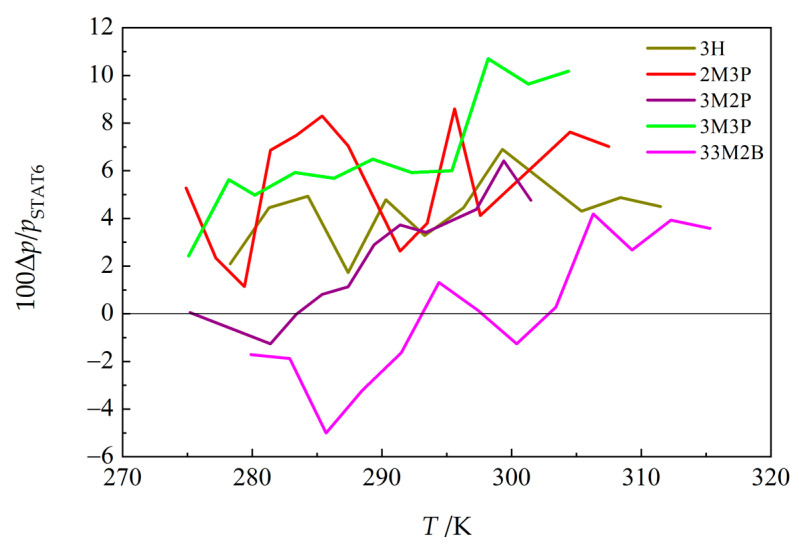
Relative deviations of vapor pressures reported by Kulikov et al. [32] from values *p*_STAT6_ obtained in this work (Table S2 in the Supplementary Materials). Δ*p* = (*p* [32] − *p*_STAT6_). See Table 5 for compound abbreviations.

Kulikov et al. [32] used the transpiration method to determine vapor pressures for 3H, 2M3P, and three additional compounds studied here (3M2P, 3M3P, and 33M2B). The purities were >0.995 mole fraction, and traces of water from the samples were reportedly removed by flashing them with nitrogen before use. A correction for the residual vapor pressure at the temperature of the cold trap (243 K) was applied by the authors. Similarly to Thomas, temperatures were reported to one decimal place. Data published by Kulikov et al. [32] tend to be higher than ours (by up to 10 percent), and appear to have a different slope and significantly greater scatter (see Figure 3).

Similar observations were made previously for the same laboratory, where noticeable differences between transpiration results and static measurements from other groups (e.g., Rostock, Utrecht, and Lyon) were reported and illustrated in Figures 11, 13, and 17 of Zaitsau et al. [42].

### 3.2. Simultaneous Correlation of Vapor Pressures and Related Thermal Data (SimCor)

Since arc-plot graphs [28] cannot assess the thermodynamic consistency of data but only highlight differences between individual data sets, the next step was to simultaneously correlate vapor pressures *p* and related thermal data, namely, enthalpies of vaporization $\Delta_{l}^{g}H_{m}$ (available only for 2M2P [43]), and $\Delta_{l}^{g}C_{p,m}^{0}$, the difference between the ideal-gas (Section 2.3) and liquid heat capacities [21] (SimCor method, Section 4.4). The results confirm that the vapor pressures obtained in this work using the static method are reasonably consistent with BSU ebulliometric data, as well as with $\Delta_{l}^{g}H_{m}$ and $\Delta_{l}^{g}C_{p,m}^{0}$. Thus, for 3H, 2M2P, and 2M3P, only the data from this work and those reported by BSU (given in bold in Table 2) were used to determine the parameters of the Cox equation, Equation (3), reported in Table 3. This is the same way the SimCor method was used in our previous works. This approach, however, could not be used for the remaining five compounds, for which no reliable vapor-pressure values are available at pressures higher than those achievable with our STAT6 instrument (approx. 1 kPa, see Table 2).

**Figure 4:**
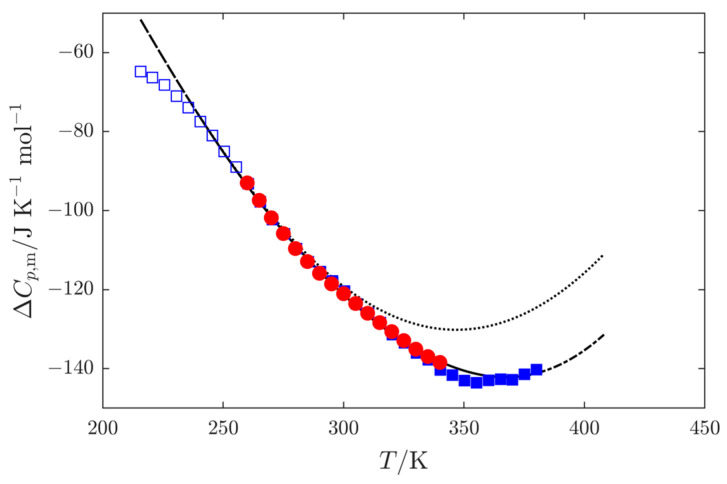
Heat-capacity difference $\Delta_{l}^{g}C_{p,m}=C_{p,m}^{g}-C_{p,m}^{l}$ typical for aliphatic alcohol (3M2P in this case) based on liquid heat capacities $C_{p,m}^{l}$ reported previously [21] and gas heat capacities $C_{p,m}^{g}$, obtained from $C_{p,m}^{g0}$ (Section 2.3) and a correction for gas-phase non-ideality (see Section 4.4). 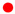, $C_{p,m}^{l}$ from SETARAM Microcalvet calorimeter; 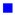, 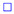 $C_{p,m}^{l}$ from Perkin Elmer 8500 calorimeter; 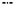, $\Delta_{l}^{g}C_{p,m}$ from the SimCor procedure; 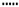, $\Delta C^{\prime }=C_{p,m}^{g0}+[pVT\;\mathrm{correction}]$ from the SimCor procedure (see Equation (S7)).

Extrapolating vapor pressures measured at low temperatures toward higher temperatures is always very uncertain, and this is even more true for alcohols due to their special behavior influenced by hydrogen bonding. For most compounds, the temperature dependence of $\Delta_{l}^{g}C_{p,m}^{0}$ is more or less linear with a positive slope (see, e.g., data for biphenyl and diphenyl methanone in Růžička and Majer [44], or for several *n*-alkanes in [45]). In the case of alcohols, $\Delta_{l}^{g}C_{p,m}^{0}$ exhibits a complex, highly nonlinear dependence on temperature, showing a negative slope at lower temperatures, then passing through a minimum and changing to positive values at higher temperatures (see, e.g., Figure 4 for 3M2P or data for 1-octanol in [44]). Extrapolated data from the low-temperature region would therefore follow the negative slope of $\Delta_{l}^{g}C_{p,m}^{0}$ and lead to unrealistic values above the temperature corresponding to the $\Delta_{l}^{g}C_{p,m}^{0}$ minimum. Because there is an exact thermodynamic relation between vapor pressure and $\Delta_{l}^{g}C_{p,m}^{0}$ (see Equation (S4) in the Supplementary Materials), this also means that extrapolated vapor pressures would be subject to greater errors than for other compound types.

Since we previously reported liquid heat capacities up to 380 K [21] and calculated ideal-gas heat capacities for all compounds in this study (Section 2.3), there is a possibility to simultaneously correlate the vapor pressures of this work (available up to 308 K; see Table 2) with $\Delta_{l}^{g}C_{p,m}^{0}$. The uncertainty of the data obtained using this thermodynamically controlled extrapolation is influenced by the uncertainty in the *pVT* correction for non-ideal gas-phase behavior (see Section 4.4). This correction increases with temperature, and its evaluation requires an estimation method for the second virial coefficient [46], which in turn requires either experimental [47] or estimated [48,49] values of critical temperatures and pressures.

Although the vapor-pressure values obtained in this way are likely to have higher uncertainty than ebulliometric values from reputable laboratories, they are considered more reliable than *p* values derived from a simple extrapolation of low-pressure data. The resulting uncertainty of the vapor pressure *p* and $\Delta_{l}^{g}H_{m}$ obtained in this way, affected by the use of estimated properties, is estimated to be less than 1000 Pa for *p* and 2 percent for $\Delta_{l}^{g}H_{m}$ at 380 K (vapor pressures at this temperature are in the range from 20 to 40 kPa for the hexanols of this study).

Note that, due to the complex temperature dependence of $\Delta_{l}^{g}C_{p,m}^{0}$, the Cox equation (Equation (3)), must be used with four parameters (see Table 3). 

**Table 3:** Final parameters of the Cox Equation (Equation (3)).

Compound	Phase	(*T*_min_−*T*_max_)/K ^a^	*T*^0^/K	*p*^0^/Pa	*A*_0_	*A*_1_·10^3^	*A*_2_·10^6^	*A*_3_·10^9^	*σ*, 100*σ*_r_ ^b^
3H	l	238–409	408.52	100,000	2.7943507 ± 0.0059517	2.2516151 ± 0.0651354	−12.252175 ± 0.23786466	12.970439 ± 0.2937654	59.9 Pa, 0.56
2M2P	l	238–397	395.4686	100,000	2.7693308 ± 0.01399173	2.1093639 ± 0.15207155	−10.943814 ± 0.55098098	10.504174 ± 0.6701256	174.3 Pa, 0.30
2M3P	l	233–400	400.8863	100,000	2.6780467 ± 0.01883502	3.3340287 ± 0.20516587	−16.41784 ± 0.7460045	17.757143 ± 0.9121549	269.6 Pa, 0.63
3M2P		238–381	407.3302	100,000	2.8662138 ± 0.004850913	1.2889432 ± 0.05356992	−8.9337753 ± 0.19629249	9.441982 ± 0.2440539	0.347 Pa, 0.48
3M3P	l	233–381	395.7459	100000	2.5391802 ± 0.00984653	4.7073126 ± 0.1078361	−21.083236 ± 0.39316663	22.884878 ± 0.4827427	0.103 Pa 0.22
	crI	215–250	346.0881	100,000	3.2357548 ± 0.00206600	−0.2490463 ± 0.00813569	0	0	0.018 Pa, 0.50
22M1B+	l	251–381	408.3389	100,000	2.7486585 ± 0.00918374	2.2959203 ± 0.1013443	−11.991292 ± 0.37115292	13.142867 ± 0.4613495	0.337 Pa 0.07
	crI	238–252	377.6666	100,000	2.9942036 ± 0.00088889	0	0	0	0.006 Pa 0.41
23M2B	l	253–381	390.6690	100,000	2.5801146 ± 0.01162609	4.1167729 ± 0.12632841	−18.705154 ± 0.45832402	20.113871 ± 0.5584105	0.364 Pa 0.07
	crI	215–262	352.6666	100,000	3.1640758 ± 0.00205365	−0.29312593 ± 0.00933850	0	0	0.044 Pa 0.39
33M2B	l	265–381	394.1552	100,000	2.7453941 ± 0.01596937	2.2111073 ± 0.17130408	−12.379211 ± 0.6142143	13.252975 ± 0.7411688	0.268 Pa 0.14
	crI	248–279	366.4122	100,000	3.1057766 ± 0.00091999	−0.51527251 ± 0.00412352	0	0	0.176 Pa 0.31
	crII	215–258	361.3012	100,000	3.1386075 ± 0.00229197	−0.45595335 ± 0.01330643	0	0	0.058 Pa 0.82

^a^ Parameters developed by the SimCor method (Section 4.4) using vapor/sublimation pressures given in bold in Table 2, ideal-gas heat capacities (Section 2.3), and liquid heat capacities [21] are valid over the joint temperature interval of the input thermodynamic data. The uncertainty in *p* is indicated by error bars in Figure 5 for 3H, 2M2P, and 2M3P, for which ebulliometric data from BSU are available. For a discussion of the uncertainty in *p* for the remaining compounds, see the text. See Table 5 for compound abbreviations. ^b^ σ is the standard deviation of the fit defined as $\sigma ={\left[\sum_{i=1}^{n}{\left(\Delta p\right)}_{i}^{2}/\left(n-m\right)\right]}^{1/2}$, where Δ*p* is the difference between the experimental and calculated values, *n* is the number of experimental points used in the fit, and *m* is the number of adjustable parameters of the Cox equation (Equation (3)). σ_r_ is the relative standard deviation of the fit defined as $\sigma_{r}={\left[\sum_{i=1}^{n}{\left(\Delta \ln p\right)}_{i}^{2}/\left(n-m\right)\right]}^{1/2}$.

**Figure 5:**
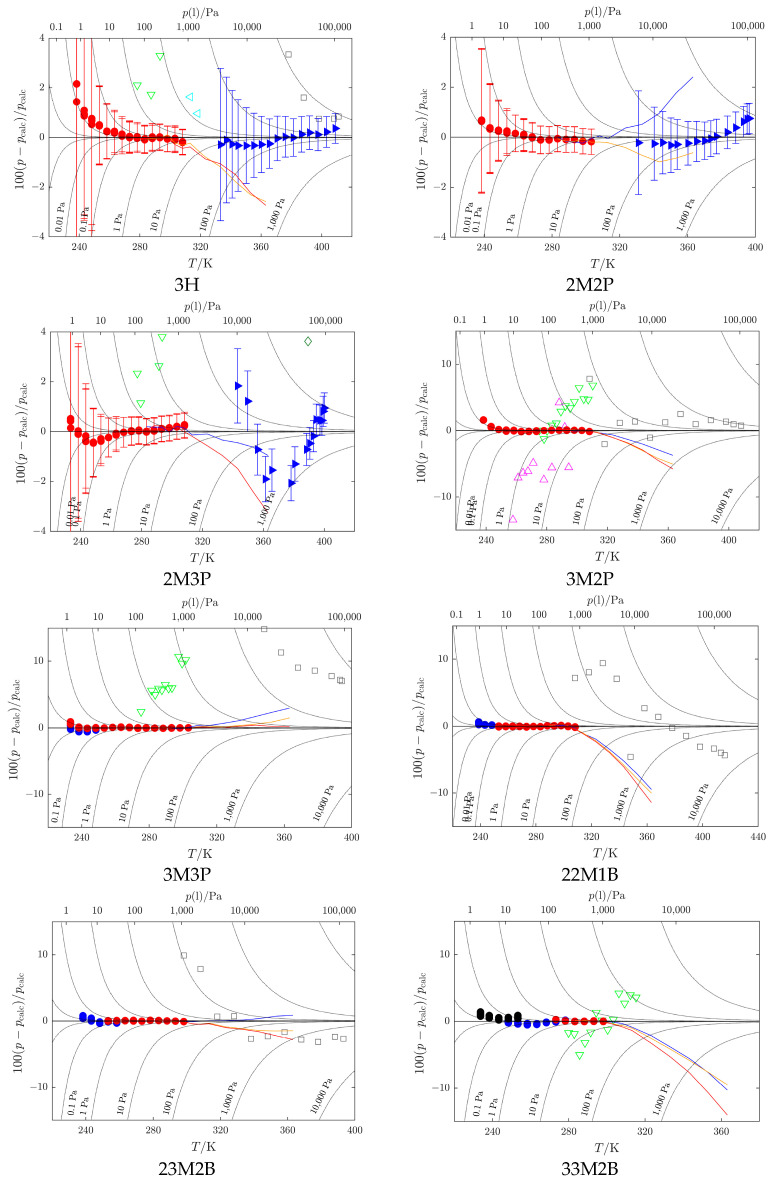
Deviation plots for vapor pressure (see Table 5 for compound abbreviations). *p*_calc_ is calculated by means of the Cox equation (Equation (3)), with parameters presented in Table 3. This work: 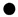 Cr II, 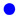 Cr I, 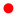 liquid; 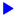 Belarus State University—BSU (3H: Sachek et al. [18]; 2M2P: Markovnik et al. [26]; 2M3P: Brazhnikov et al. [17]); 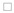 Hovorka et al. (3H: [33], 2M2P: [34], 2M3P and 3M3P [35], 3M2P and 23M2B [36], 22M1B [38]); 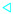 N’Guimbi et al. [20]; 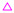 Thomas et al. [31]; 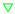 Kulikov et al. [32]; 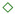 Loginova et al. [40]. Vapor pressures obtained by a combination of static and indirect chromatographic methods (GLC-ACRT (Activity Coefficients–Retention Times) method, Section 3.5 and Section 4.6): **−** reference 2M2P, **−** reference 2M3P, **−** reference 3H. Data sets represented by filled symbols were used in the SimCor procedure. For compounds for which ebulliometric data from BSU are available (3H, 2M2P, 2M3P), error bars are given to show data uncertainty.

Deviations of individual vapor-pressure data points from values obtained by the SimCor procedure are shown in Figure 5.

### 3.3. Sublimation Pressures

For 3M3P, 22M1B, 23M2B, and 33M2B, sublimation pressures were also determined for the Crystal I phase, and for 33M2B also for the Crystal II phase. No data for comparison were found in the literature, and structural data for these compounds were not available in the Cambridge Structural Database [50]. Short temperature ranges (typically 10 to 30 K; see Table 2) together with low measured pressure values allowed only the parameters of the simplest equation, ln *p* = *A* − *B*/*T*, to be determined, which assumes a constant value of the sublimation enthalpy.

To overcome this limitation, simultaneous correlation of the sublimation pressures with the heat-capacity differences (Section 2.3 and our previous work [21]) and phase-transition enthalpy values [21] was performed, allowing the parameters of the Cox equation (Equation (3)) to be determined, although a smaller number of parameters was sufficient for the description (see Table 3). For 33M2B, heat capacities measured with the power-compensation differential scanning calorimeter (PC-DSC) between 216 and 241 K were labeled as crI, although crI should reversibly transform to crII below approx. 258 K based on the DSC experiments. Note that it is not possible to verify crystal-phase identity in the DSC by any means other than through its phase-transformation properties. The relatively broad temperature range, where the heat capacities could not be evaluated in [21], compared to other compounds, might suggest a series of transformations, i.e., crII-crI followed by crI-l. In the SimCor procedure, the discussed data were considered to belong to crII.

Table 4 compares phase-transition temperatures and enthalpies resulting from the SimCor procedure with the respective literature values. The thermodynamic consistency of the calorimetric data [21] and vapor-pressure measurements proves to be reasonable. All phase-change enthalpies are described within their uncertainty and the phase-change temperature within 1 K. Negative deviations of the DSC-determined melting temperatures [21] somewhat exceeding the experimental uncertainties can be observed for the compounds with lower purity: 23M2B (*x* = 0.9946) and 33M2B (*x* = 0.9967). Although the DSC values were corrected for impurity content [21], the estimated correction may be underestimated since the onset temperature is more sensitive to impurity content than the ideal-solubility equation suggests. On the other hand, the DSC value for the crII-crI transition temperature of 33M2B [21] shows a slight positive deviation from the correlation, which agrees with the fact that the transition can overheat in the DSC experiments for kinetic reasons, while the intersection of vapor-pressure curves is not susceptible to such effects.

### 3.4. Enthalpies of Vaporization

Vaporization enthalpies can be derived via the Clapeyron equation using the Cox equation (Equation (3)), with parameters presented in Table 3, and applying corrections for non-ideality of the gas phase (see Section 4.4). The resulting values are given in Table S7 in the Supplementary Materials. Calorimetric enthalpies of vaporization suitable for comparison are available only for 2M2P (Majer et al. [43]) in the temperature range from 298 K to 368 K. Agreement with the data derived in this work is excellent, within 0.3 percent.

**Figure 6:**
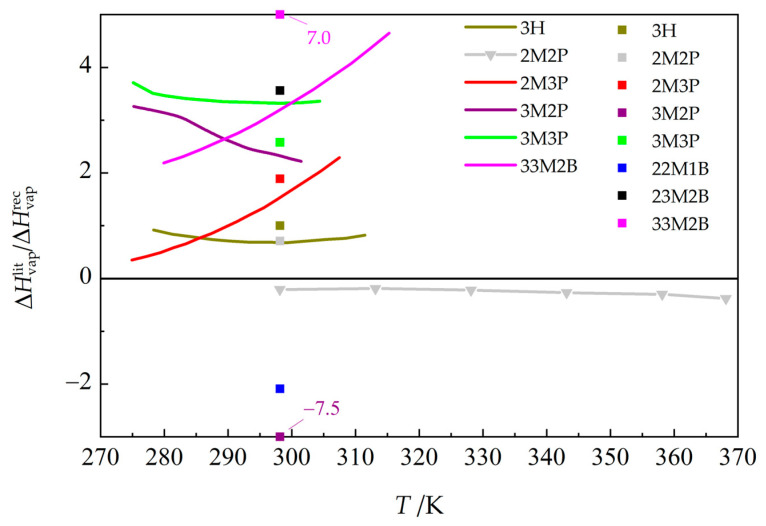
Relative deviations of the literature vaporization enthalpies $\Delta H_{\mathrm{vap}}^{\mathrm{lit}}$ (lines: Kulikov et al. [32], derived from vapor pressures; 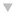 Majer et al. [43], calorimetry) and estimated values (filled squares, group-contribution method by Kolská et al. [51]) from the results of this work, $\Delta H_{\mathrm{vap}}^{\mathrm{rec}},$ derived from the Cox equation (Equation (3)), with parameters from Table 3 (resulting vaporization enthalpies, including corrections for non-ideality applied in their calculation, are given in Table S7 in the Supplementary Materials). See Table 5 for compound abbreviations.

Vaporization enthalpies were also derived from vapor pressures by Kulikov et al. [32]; the authors neglected the non-ideality of the gas phase, which is adequate for the temperature range of their measurements. The differences are positive for all five compounds measured by both Kulikov et al. [32] and this work, spanning from 0.35 percent to 4.65 percent, as shown in Figure 6.

Another source of vaporization enthalpies is the estimation method developed by Kolská et al. [51]. This method is based on the IUPAC compilation of critically evaluated calorimetric vaporization enthalpies [52]. As the number of vaporization enthalpies for branched aliphatic alkanols covered by this compilation is rather limited, it could be expected to be less reliable for this class of compounds, which is confirmed in Figure 6.

**Figure 7:**
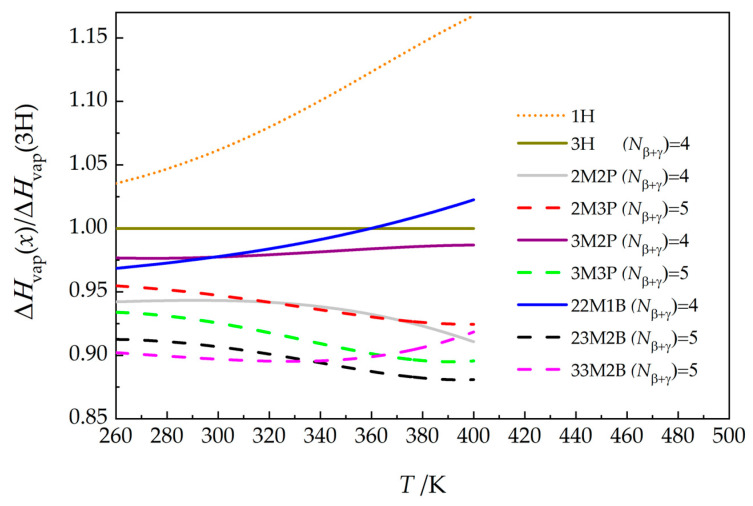
Vaporization enthalpies for hexanols of this work relative to that for 3H (see Table 5 for compound abbreviations). Values for 1-hexanol [22] are shown for comparison. Values used to construct this graph are given in Table S7 in the Supplementary Materials.

Based on the recommended values of this work (Table S7 in the Supplementary Materials), it is also possible to compare the vaporization enthalpies of 3H with the remaining hexanols studied in this work as a function of temperature. Results are presented in Figure 7. It is clear that any conclusions based solely on values at 298.15 K are incomplete and might be misleading. For example, Benson [53] concluded that alcohols form tetramers held together by H-bonds (for both 1-alkanols and branched alcohols, and even for cyclohexanol) based on the constant difference between the vaporization enthalpies of alcohols and their hydrocarbon homomorphs at 298.15 K. However, Majer et al. [43] showed that these differences are not constant at higher temperatures. Our previous studies of aliphatic heptanols [54] and octanols [55] clearly showed that alcohols in the liquid phase exist as a mixture of monomers, dimers, and higher oligomers, with the ratio of these species changing with increasing temperature in favor of simpler species.

The apparent irregularities in the temperature dependence of vapor enthalpies for 22M1B and 33M2B (see Figure 7) can be logically explained by examining the temperature dependence of the heat-capacity differences between gas and liquid $\Delta_{l}^{g}C_{p,m}^{0}$ for these compounds (this trend is due to the small increase in heat capacity with temperature for these two substances; see Figure 5 in [21]).

Large differences in vapor pressures (Figure 1a), liquid heat capacities (Figure 1b), and vaporization enthalpies (Figure 7) for compounds of the same overall formula demonstrate that it is difficult to use conventional methods to estimate the contribution of the alcohol group as a function of temperature; it may therefore be necessary to use an unconventional approach that takes into account the changing ratio of oligomers with temperature.

It should be noted that methods for estimating the heat capacity of liquids [56,57] were also unsuccessful in the case of branched heptanols (Section S3 in the Supporting information of [54]) and octanols (Section S3 in the Supporting information of [55]), even though they were developed using a significantly higher number of critically evaluated data [58,59,60] than those used for vaporization enthalpies.

### 3.5. Vapor Pressures Obtained by Combining Static and Indirect Chromatographic Methods

**Figure 8:**
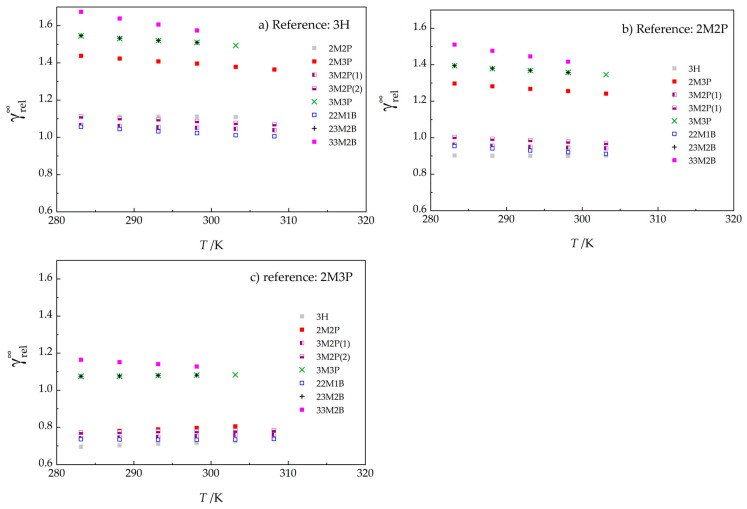
The ratio of activity coefficients $\gamma_{\mathrm{rel}}^{\infty }$ in Equation (2) for the compounds studied, with (**a**) 3H, (**b**) 2M2P, and (**c**) 2M3P as reference compounds. For 3M2P, which is a mixture of diastereomers, chromatography yields two distinct retention times. Values shown in this figure are given in Table S8 in the Supporting Materials. See Table 5 for compound abbreviations.

As already mentioned in Section 2.2, there is a relationship between adjusted chromatographic retention times and vapor pressures of the reference and test compounds. The ratio of activity coefficients $\gamma_{\mathrm{rel}}^{\infty }(T)$ in Equation (1) is usually unknown, and many previous studies have assumed this ratio to be constant and equal to 1 [61,62,63,64]. Although frequently applied, this simplification introduces perhaps the largest error in the method, particularly when GLC is used to determine vapor pressures for polar substances [65]. Our calculations of $\gamma_{\mathrm{rel}}^{\infty }$ according to Equation (2):

(2) $$\frac{\gamma_{r}^{\infty }(T)}{\gamma_{x}^{\infty }(T)}=\gamma_{\mathrm{rel}}^{\infty }(T)=\frac{t_{x}^{\prime }(T)\cdot p_{x}(T)}{t_{s}^{\prime }(T)\cdot p_{r}(T)}$$

in the temperature range overlapping with static measurements, and shown in Figure 8 for compounds in this study using 3H, 2M2P, and 2M3P as the reference compounds, corroborate this view.

Values of $\gamma_{\mathrm{rel}}^{\infty }$ were calculated using the adjusted retention times from Table 1 and vapor pressures calculated via the Cox equation (Equation (3)), with parameters given in Table 3. Values of $\gamma_{\mathrm{rel}}^{\infty }$ range from 1.0 for 22M1B to 1.68 for 33M2B (Figure 8a), calling into question the validity of assuming $\gamma_{\mathrm{rel}}^{\infty }=\;\gamma_{r}^{\infty }/\gamma_{x}^{\infty }=1$ (and values based on this simplification), and confirming our previous findings [23]. When 2M2P and 2M3P are used as the reference compounds (r), values of $\gamma_{\mathrm{rel}}^{\infty }$ vary from 0.90 to 1.51 and from 0.73 to 1.13, respectively (see Figure 8b,c, and also Table S8). Figure 8 shows that a linear model appears capable of representing the temperature dependence of $\gamma_{\mathrm{rel}}^{\infty }$ in the overlapping temperature range 283 K to 308 K with reasonable accuracy. Note that 3H, 2M2P, and 2M3P were selected as reference compounds because their vapor pressures are known up to 363 K (the maximum temperature of indirect chromatographic measurements in this work) and even higher (up to the normal boiling point), thus allowing validation of the extrapolation procedure based on combining the static and chromatographic methods, assuming linear dependence of $\gamma_{\mathrm{rel}}^{\infty }$ on temperature (Equation (4); parameters of this equation are given in Table S9 in the Supplementary Materials).

**Figure 9:**
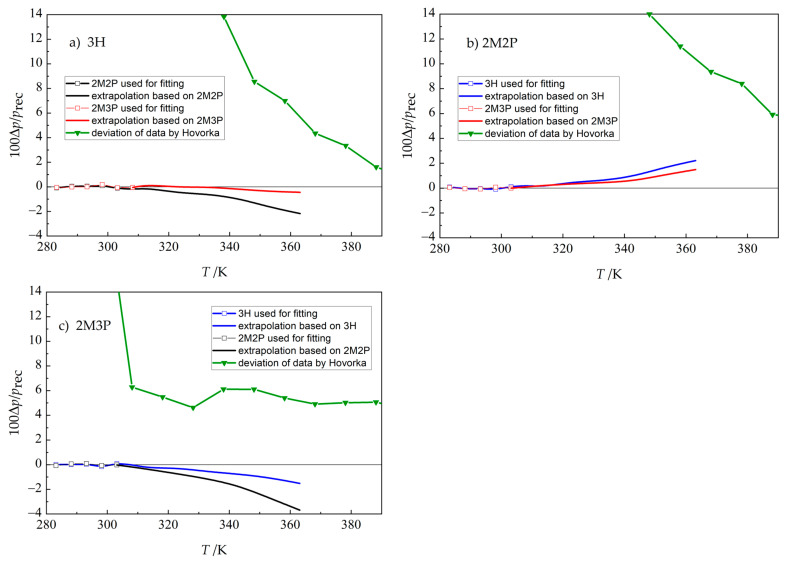
Relative error in vapor-pressure values based on extrapolation (GLC-ACRT method combining static measurements (283 K to 308 K) and chromatographic data (283 K to 363 K); see Section 3.5 and Section 4.6). Δ*p = p*(GLC-ACRT) − *p*_rec_, where *p*_rec_ is calculated from the Cox equation (Equation (3)) using parameters from Table 3. Relative errors of vapor pressures published by Hovorka (3H: [33]; 2M2P: [34]; 2M3P [35]) from the recommended *p*_rec_ values are shown for comparison. See Table 5 for compound abbreviations.

A comparison of the extrapolated (to 363 K) vapor-pressure values obtained using 3H, 2M2P, and 2M3P as references with directly measured data reported by BSU [17,18,30] is shown in Figure 9, together with older directly measured data by Hovorka (3H: [33]; 2M2P: [34]; 2M3P [35]). It can be seen that the values of *p* obtained by our method deviate in all cases by less than 4% for those three compounds in the given temperature range. Note, however, that uncertainty might be higher in cases when the overlap of temperature ranges between the static and chromatographic methods was short and the parameters of the linear fit (Equation (4)) were determined from only four datapoints.

Generally, we consider uncertainties of vapor pressures obtained this way to be higher than those obtained by the SimCor method, described in Section 3.2; the differences between the two methods are within ca. 1000 Pa (4 percent or less) for all compounds except for 22M1B and 33M2B (errors more than 10 percent, ca. 2 kPa for 22M1B, and 4 kPa for 33M2B). The result for 33M2B is likely associated with the fact that parameters of Equation (4) for the temperature dependence of $\gamma_{\mathrm{rel}}^{\infty }$ were established from only four datapoints. The reason for disagreement in the case of 22M1B remains unclear. On the other hand, determining GLC retention times is far less demanding than experimentally determining liquid heat capacities together with calculating ideal-gas heat capacities required by the SimCor method. Also, the results of this GLC-ACRT method are still better than those of the best available estimation method for vapor pressures developed by Rarey and co-workers [49,66,67], which at 363 K deviate from the SimCor results (Section 3.2) by up to 36 percent (only 2M3P differs by less than 5 percent). See Section S5 in the Supplementary Materials for more information. Note that this estimation method [66], although outperforming other estimation methods according to its authors, does not perform well even for 1-alkanols if a wider temperature/pressure range is considered (see detailed comparison in Figure S5 of our previous paper [22]).

### 3.6. Discussion of Trends in Thermodynamic Properties of the Studied Hexanols

In our previous work [21], we reported experimental liquid heat capacities and theoretical results (obtained from molecular-dynamics simulations) for a group of aliphatic hexanols. The theoretical parameters included the intensity of the first peak in the radial distribution function *g*_HO_ of hydroxyl H^…^O contacts in the bulk liquid, the radial distribution function itself, the average coordination number of hydroxyl H…O contacts within the first coordination sphere, and the cohesive energies. The results clearly demonstrated that the sum of the numbers of β- and γ-carbon atoms (*N_β_*_+*γ*_) is an important factor governing the behavior of these compounds. However, the interpretation was somewhat limited by the relatively small differences in the heat capacities among individual hexanols and by the fact that the theoretical calculations were performed only at 350 K.

The present study provides further insight into the structure–property relationships within this group of compounds. The role of the *N_β_*_+*γ*_ parameter was confirmed, as can be seen in Figure 1a and Figure 7; compounds with *N_β_*_+*γ*_ = 5 generally have higher vapor pressures and lower vaporization enthalpies than compounds with *N_β_*_+*γ*_ = 4.

The experimental data obtained here can serve as benchmark values for future theoretical calculations, which—in the case of vapor pressures—are often subject to large uncertainties even for non-hydrogen-bonded compounds.

## 4. Materials and Methods

The molar masses of the compounds were calculated based on recommendations by IUPAC [68], the molar gas constant was R = 8.314462618 J K^−1^ mol^−1^ [69], and temperatures are based on the International Temperature Scale ITS-90 [30].

### 4.1. Sample Description

The studied alcohols were the same as those used in our previous paper [21]; they were either of commercial origin or were synthesized in our previous work. For the reader’s convenience, the information is repeated here. Samples with a molar fraction below 0.99 were purified using spinning-band distillation and then stored over 0.4 nm molecular sieves. All subsequent sample manipulations were performed in a dry box under a dry nitrogen atmosphere. The sample purities were checked by gas–liquid chromatography and water content was determined using Karl Fischer analysis. All information about the samples used in this work is reported in Table 5.

**Table 5:** Sample description.

Compound	Abbreviation	CAS RN	Supplier	Original Molar Fraction ^a^	Final Molar Fraction ^b^	Water Mass Fraction *w*_H2O_ (·10^−6^) ^c^
(±)-3-hexanol	3H	623-37-0	Aldrich (Burlington, MA, USA)	0.994	0.9990 ^d^	28.8
2-methyl-2-pentanol	2M2P	590-36-3	Aldrich	0.997	0.9982	12.8
(±)-2-methyl-3-pentanol	2M3P	565-67-3	Aldrich	0.994	0.9992 ^d^	26.0
(±)-3-methyl-2-pentanol ^e^	3M2P	565-60-6	TCI (Tokyo, Japan)	0.99	0.9998	14.8
3-methyl-3-pentanol	3M3P	77-74-7	TCI	0.996	0.9989 ^d^	20.9
2,2-dimethyl-1-butanol	22M1B	1185-33-7	Synthesized ^f^ (London, UK)	-	0.9993 ^d^	36.0
2,3-dimethyl-2-butanol	23M2B	594-60-5	Aldrich	0.993	0.9946	34.0
(±)-3,3-dimethyl-2-butanol	33M2B	464-07-3	Aldrich	0.989	0.9967	16.6

^a^ From the certificate of analysis supplied by the manufacturer, determined by gas–liquid chromatography (GLC). ^b^ Gas–liquid chromatography analysis by a Hewlett-Packard 6890 gas chromatograph equipped with an HP5 cross-linked 5% PHME siloxane column, length 30 m, film thickness 0.25 µm, i.d. 0.32 mm, and an FID detector. ^c^ All samples were dried using molecular sieves and kept in a glovebox (MBraun LabStar) to avoid contamination by moisture. Water content was determined by Karl Fischer analysis (Metrohm 831). ^d^ Samples were purified by spinning-band distillation (B/R Instrument). ^e^ Mixture of diastereomers. ^f^ Synthesis described previously [21].

### 4.2. Vapor Pressures

The vapor-pressure measurements were performed using an apparatus with capacitance diaphragm gauges STAT6 [70]. As this apparatus has been previously described in detail [70], we present here only its operating pressure range (0.5–1333 Pa) and temperature range (233–308 K). The reader is referred to the original paper for details on its design, calibration, and measurement procedure. The resulting combined expanded uncertainty of the vapor-pressure measurements (0.95 level of confidence, *k* = 2) is adequately described by the expression *U*_c_(*p*/Pa) = 0.005(*p*/Pa) + 0.05. The measurements were performed by varying the temperature at random to detect systematic errors caused by insufficient degassing of the sample. The experiments were carried out repeatedly at selected temperatures. When the pressure did not change with the number of measuring cycles, the sample was considered completely degassed (typically after hundreds of measuring cycles), and the final set of data was recorded.

### 4.3. Thermodynamic Properties in the Ideal-Gas State

We previously calculated ideal-gas properties of 1-alkanols, including 1-hexanol, using the R1TM approach [22]. Comparison with the experimental values for the lower 1-alkanols showed agreement well within 1%. The R1TM approach combines carefully selected quantum chemistry calculations and three statistical-mechanics models: (i) the rigid-rotor–harmonic oscillator (RRHO) approximation; (ii) corrections for terminal (and other symmetrical) top rotations using the one-dimensional hindered-rotor approximation (1DHR) [71]; and (iii) the population of conformers based on the Boltzmann distribution.

The calculations in this work (optimization of molecular geometries, calculation of fundamental vibrational frequencies, and potential-energy scans) were performed using density functional theory (DFT) at the B3LYP/6-311 + G(2df,p) level of theory with the empirical dispersion correction D3 developed by Grimme et al. [72], as implemented in the Gaussian 16 software package [73]. Calculated vibrational frequencies were scaled by the double-linear scaling factor (SF) optimized on *n*-alkanes [74]: SF(*ν* > 2000 cm^−1^) = 0.960; SF(*ν* < 2000 cm^−1^) = 0.9948 − 1.35∙10^−5^ *ν*_i_.

Potential-energy profiles of internal rotations were obtained using a relaxed scan starting from the lowest-energy conformer, and their contributions to thermodynamic properties were obtained by solving a one-dimensional Schrödinger equation using the FGH method [75]. As observed previously [22], rotational barriers of the hydroxyl groups are too low to describe these degrees of freedom using the RRHO model with a correction for mixing. Therefore, from each set of conformers that differ only in the rotation of the hydroxyl group, only the most stable one was included in the mixing model. Thus, the conformers treated in the R1TM approach represent one-third of all stable nonisomorphic conformers.

### 4.4. Simultaneous Treatment of Vapor Pressures and Related Thermal Data (SimCor Method)

The simultaneous correlation of vapor pressures and related thermal properties (SimCor, suggested in a simplified form by King and Al-Najjar [76]) is based on exact thermodynamic relationships, and the procedure must therefore yield reliable results, provided that the input data are of reasonable accuracy. A great advantage of this approach is that a single equation can furnish a description of the temperature dependences of several thermodynamic properties, resulting in a set of vapor-pressure equation parameters that are valid over a combined temperature range of all input experimental values. The SimCor method also provides a test on the consistency of different experimental data (vapor pressures *p*, calorimetric vaporization enthalpies, and differences in the heat capacities between the ideal-gas and liquid phases, where and were obtained as described in the previous section and from the calorimetric measurements, respectively). The SimCor method has been described in detail in, e.g., in [44,45], and is summarized in Section S3 of the Supplementary Materials for the reader’s convenience. It was used in our laboratory to develop recommended data on vapor pressure and thermophysical properties for several groups of crystalline and liquid compounds (see, e.g., [22] and references therein). The real behavior of the gas phase was approximated using the method of Tsonopoulos [46], employing dipole moments and critical temperatures and pressures.

The Cox equation [77] was used within the SimCor procedure to describe the vapor pressures and the linked thermodynamic properties, since it requires a lower number of adjustable parameters than other equations while providing a comparable description. This equation was found to be adequate for simultaneously describing vapor pressure and related thermal data as a function of temperature over an extended pressure range, down to the triple point [44]:

(3) $$\ln\frac{p}{p^{0}}=\left(1-\frac{T^{0}/K}{T/K}\right)\exp\left(\sum_{i=0}^{N}A_{i}{\left(T/K\right)}^{i}\right)$$

where *p*^0^ and *T*^0^ are the reference pressure and temperature, respectively, and *A_i_* are the adjustable parameters, the number of which depends on the length of the temperature range of the data involved in the SimCor procedure and on the type of compound considered.

### 4.5. Gas–Liquid Retention Time Determination

The retention times of all compounds were determined using an Agilent Technologies 6890N gas chromatograph (Agilent, Santa Clara, CA, USA) equipped with a flame ionization detector (250 °C), electronic pneumatic control (EPC), a split/splitless injection port (200 °C), an HP 7863B automatic injector, and GC ChemStation software Rev. A.09.01 [1206] (Agilent, Santa Clara, CA, USA). The chromatograph was equipped with a cryogenic cooling system using liquid nitrogen to enable cooling of the chromatographic oven below laboratory temperature. All measurements were performed on a nonpolar dimethylpolysiloxane bonded-phase fused-silica capillary column (Agilent J&W Ultra 1, 20 m × 32 μm i.d., 0.52 μm film thickness, (Agilent, Santa Clara, CA, USA).

Isothermal measurements were performed in the temperature range from 283 K to 363 K with a split ratio of 1:60 and a nitrogen head pressure of 25 kPa (EPC). Measurements were carried out with 30 min of temperature conditioning between runs. The standard uncertainty in the sample-temperature measurements was *u*(*T*) = 0.05 K. The alcohols were injected into the inlet as carbon disulfide solutions (0.2 µL). Symmetrical peaks indicated that infinite dilution was achieved for all the distribution processes. Three compounds for which reliable vapor pressures up to *T*_nbp_ exist (3H, 2M2P, and 2M3P) were used as reference compounds. Adjusted retention times were calculated by subtracting the retention time of methane from the retention time of the analyte. All retention times used for calculations were the means of three separate runs. The relative standard deviation (RSD) was ≤0.05% for short retention times, i.e., at temperatures above approximately 323 K; at lower temperatures, the RSD gradually increased and was approximately ≤ 0.5% at the lowest temperature of 283 K.

### 4.6. Indirect GLC-RT Method for Determination of Vapor Pressures

As Equation (1) shows, gas-chromatographic vapor-pressure determination can only yield relative values, so reference data are needed for a transformation into absolute vapor pressures. The term indirect refers to the procedural properties of the measurement. Hence, all GLC-RT methods based on Equation (1) are called indirect because they do not directly measure the vapor pressure but instead use correlations with chromatographic retention times. In principle, these methods are based on correlating the retention time of the test compound with that of a reference compound whose vapor pressures are known (measured with a suitable direct method) at the temperatures used, thus allowing prediction of the vapor pressure of the test compound [78,79].

In this work, starting from Equation (1) and the original suggestion by Orbey and Sandler [80], we proposed a relative measurement technique for the infinite-dilution activity-coefficient ratio using the GLC method. Unlike works exploring a single reference and neglecting the activity-coefficient ratio (GLC-RT1S) [23] to determine vapor pressures, our model integrates the temperature-dependent vapor pressure data determined by the static method with the corresponding retention-time data obtained by the GLC technique.

We hypothesized that the temperature-dependent ratio of activity coefficients from Equation (2) in the overlapping temperature region of the static and chromatographic methods (283 K to 308 K) can be used to extrapolate it toward higher temperatures, provided an acceptable correlation exists.

Based on our previous work [23], it can be expected that, in the temperature range where static and chromatographic measurements overlap, a linear correlation represented by Equation (4)

(4) $$\frac{\gamma_{r}^{\infty }(T)}{\gamma_{x}^{\infty }(T)}=\gamma_{\mathrm{rel}}^{\infty }(T)=a+bT$$

appears to be suitable for describing this dependence (*a* and *b* are the regression coefficients, and *T* is temperature (K)).

Equation (4) then enables extrapolation to temperatures exceeding the original temperature ranges. Hence, for extrapolated values of and measured $t_{x}^{\prime }\left(T\right),\;t_{r}^{\prime }(T)$, and $p_{r}\left(T\right),$ it is possible to evaluate the unknown $p_{x}(T)$. The vapor pressures obtained in this way are indicated in the text by the acronym GLC-ACRT (Activity Coefficients–Retention Times).

It is clear that the linearity assumed by Equation (4) may not hold over a wide range of temperatures, so that the extrapolated values obtained in this way are subject to increasing error as the length of the extrapolation increases. Extrapolation is also less reliable when the temperature range in which data from both the direct method (in this case, the static method) and the chromatographic method are available is too narrow—for example, when the saturated vapor pressure already exceeds the measurement range of the static apparatus gauge. It is also worth mentioning again that chromatographic measurements are relative, and that retention times are also influenced by the condition of the chromatographic column, which changes over time. Unlike measurements performed by the static method, it is therefore recommended to inject both test and reference compounds simultaneously into the column and to measure all retention data within a relatively short time interval. Otherwise, the data measured later might not fit the trend of the previously measured data set. This aspect is not usually discussed in studies employing indirect chromatographic methods for determining saturated vapor pressures; however, inconsistencies in published retention times appear to indicate that this factor plays a significant role.

## 5. Conclusions

In this work, new vapor-pressure data were determined using the static method for eight aliphatic hexanols in the low-pressure region (below approximately 1.3 kPa). Heat capacities in the ideal gaseous state were also determined for the same set of compounds.

These new data were combined with results from our previous research and selected literature sources to establish reliable vapor pressures and vaporization enthalpies over an extended temperature range, reaching higher temperatures. For three compounds (3-heptanol, 2-ethyl-2-pentanol, and 2-methyl-3-pentanol), reliable vapor pressures up to the normal boiling point were obtained and correlated with the data from this study as well as with related thermal properties.

For the remaining five compounds, two methods of extrapolating vapor pressures were investigated: one based on thermodynamically controlled extrapolation (SimCor), and another using chromatographic retention times measured at higher temperatures than those accessible by the static method. The former approach likely involves lower uncertainty, albeit at the expense of significantly greater experimental effort.

The results from both the experimental and extrapolated data presented in this study demonstrate that the currently best-performing estimation methods for vapor pressures [66], vaporization enthalpies [51], and liquid heat capacities [56,57] are unable to provide reliable values for aliphatic alcohols. This emphasizes the need for continued experimental research on this class of compounds.

The results of this work represent not only the input data needed to improve estimation methods but also reference data for potential future attempts to calculate the properties of aliphatic alkanols using theoretical methods.

## Figures and Tables

**Table 1:** Retention times of compounds studied in this work ^a^.

	HUT ^b^	3H ^c^	2M2P	2M3P	3M2P ^d^	3M2P ^d^	3M3P	22M1B	23M2B	33M2B
*T*/K	*t*/min									
283.15	1.623	34.366	17.650	26.382	31.214	32.488	20.987	33.990	16.575	18.666
288.15	1.631	26.392	14.081	20.570	24.129	25.010	16.592	26.098	13.296	14.827
293.15	1.656	20.723	11.477	16.396	19.043	19.731	13.441	20.470	10.914	12.053
298.15	1.667	16.381	9.425	13.180	15.166	15.623	10.969	16.185	9.021	9.866
303.15	1.703	13.342	7.964	10.876	12.415	12.736	9.192	13.157	7.681	8.313
308.15	1.738	11.043	6.845	9.143	10.313	10.572	7.829	10.913	6.638	7.123
313.15	1.750	9.177	5.914	7.728	8.621	8.830	6.708	9.097	5.756	6.136
323.15	1.800	6.788	4.694	5.877	6.439	6.567	5.240	6.730	4.618	4.857
333.15	1.828	5.240	3.877	4.663	5.023	5.103	4.252	5.209	3.838	3.993
343.15	1.864	4.268	3.351	3.888	4.128	4.178	3.616	4.250	3.333	3.434
353.15	1.897	3.628	2.994	3.372	3.536	3.569	3.188	3.620	2.989	3.058
363.15	1.926	3.210	2.760	3.033	3.149	3.166	2.904	3.208	2.761	2.810

^a^ The standard uncertainty in the sample-temperature measurements is *u*(*T*) = 0.05 K. Retention times used for the calculations were the means of three separate runs (relative standard deviation is less than 0.05%). See Section 4.4 for more details. ^b^ HUT stands for the hold-up time (the retention times of the unretained solute), determined using methane. ^c^ See Table 5 for compound abbreviations. ^d^ For 3M2P, which is a mixture of diastereomers, chromatography gives two distinct retention times.

**Table 4:** Description of phase-transition temperatures and enthalpies by the SimCor procedure.

Compound	Transition	*T_i_*(lit) [21] ^a^	*T_i_*(SC) ^b^	*T_i_*(lit) − *T_i_*(SC)	Δ*_i_H*(lit) [21] ^a^	Δ*_i_H*(SC) ^b^	Δ*_i_H*(lit) − Δ*_i_H*(SC)
3M3P	crI-l	250.1 ± 0.4	250.4	−0.3	10.7 ± 0.3	10.81	−0.11
22M1B	crI-l	251.2 ± 0.4	251.2	0.0	1.6 ± 0.1	1.60	0.00
23M2B	crI-l	261.6 ± 0.5	262.4	−0.8	8.4 ± 0.5	8.71	−0.31
33M2B	crI-l	278.4 ± 0.4	278.9	−0.5	6.4 ± 0.2	6.50	−0.10
	crII-crI	258.0 ± 0.5	257.6	0.4	1.96 ± 0.15	1.80	0.16

^a^ The literature values with their expanded uncertainties (*k* = 2). ^b^ Values derived using the SimCor procedure (SC). Note that the literature values listed were inputs for the SimCor procedure.

## Data Availability

The original contributions presented in this study are included in the article/Supplementary Materials. Further inquiries can be directed to the corresponding authors.

## Supplementary Materials

The following supporting information can be downloaded at: https://www.mdpi.com/article/10.3390/molecules30214287/s1: Table S1. Structures and abbreviations of the hexanols studied in this work; Section S1. Experimental values of vapor and sublimation pressures of the eight aliphatic hexanols studied in this work (Table S2); Section S2. Properties in the ideal-gas state (containing Table S3–S5); Section S3. Description of the SimCor method (containing Table S6); Section S4. Recommended vaporization enthalpies (containing Table S7); Section S5. Comparison of experimental and estimated vapor pressures (containing Figure S1); Section S6. Extrapolation of vapor pressures using the GLC-ACRT methodology (containing Tables S8 and S9).

## References

[1] Falbe J., Bahrmann H., Lipps W., Mayer D., Frey G.D. (2013). Alcohols, Aliphatic. Ullmann’s Encyclopedia of Industrial Chemistry.

[2] Kenneally C.J. (2001). Alcohols, Higher Aliphatic, Survey and Natural Alcohols Manufacture. Kirk-Othmer Encyclopedia of Chemical Technology.

[3] Hua Y. (2024). Research progress of higher alcohols as alternative fuels for compression ignition engines. Fuel.

[4] Jamrozik A., Tutak W. (2024). Alcohols as Biofuel for a Diesel Engine with Blend Mode—A Review. Energies.

[5] Gui L., Yang J., Yu G., Wu J., Meng X. (2025). Thermodynamic properties of renewable n-butanol: Experimental and modeling investigations. Thermochim. Acta.

[6] Arunan E., Desiraju Gautam R., Klein Roger A., Sadlej J., Scheiner S., Alkorta I., Clary David C., Crabtree Robert H., Dannenberg Joseph J., Hobza P. (2011). Definition of the hydrogen bond (IUPAC Recommendations 2011). Pure Appl. Chem..

[7] Arunan E., Desiraju G.R., Klein R.A., Sadlej J., Scheiner S., Alkorta I., Clary D.C., Crabtree R.H., Dannenberg J.J., Hobza P. (2011). Defining the hydrogen bond: An account (IUPAC Technical Report). Pur. Appl. Chem..

[8] Grabowski S.J. (2020). Hydrogen Bond—Definitions, Criteria of Existence and Various Types. Understanding Hydrogen Bonds.

[9] Böhmer R., Gainaru C., Richert R. (2014). Structure and dynamics of monohydroxy alcohols—Milestones towards their microscopic understanding, 100 years after Debye. Phys. Rep..

[10] Wójcik M.J., Ozaki Y. (2023). Spectroscopy and Computation of Hydrogen-Bonded Systems.

[11] Grabowski S.J. (2020). Theoretical Approaches. Understanding Hydrogen Bonds.

[12] Jabłoński M. (2023). Hydrogen Bonds. Molecules.

[13] Wilhoit R.C., Zwolinski B.J. (1973). Physical and Thermodynamic Properties of Aliphatic Alcohols.

[14] Cibulka I. (1993). Saturated Liquid Densities of 1-Alkanols from C(1) to C(10) and n-Alkanes from C(5) to C(16)—A Critical-Evaluation of Experimental-Data. Fluid Phase Equilib..

[15] Ambrose D., Ellender J.H., Sprake C.H.S. (1974). Thermodynamic properties of organic oxygen compounds XXXV. Vapour pressures of aliphatic alcohols. J. Chem. Thermodyn..

[16] van Miltenburg J.C., Oonk H.A.J., Ventola L. (2001). Heat capacities and derived thermodynamic functions of 1-octadecanol, 1-nonadecanol, 1-eicosanol, and 1-docosanol between 10 K and 370 K. J. Chem. Eng. Data.

[17] Brazhnikov M.M., Andreevskii D.N., Sachek A.I., Peshchenko A.D. (1975). Saturated vapor pressure of some secondary alcohols and calculations of heats of vaporization. Zh. Prikl. Khim..

[18] Sachek A.I., Markovnik V.S., Peshchenko A.D., Shvaro A.V., Andreevskii D.N. (1984). Vapor pressure of normal C5–C8 secondary alcohols. Khimicheskaya Promyshlennost.

[19] Hales J.L., Ellender J.H. (1976). Liquid densities from 293 to 490 K of nine aliphatic alcohols. J. Chem. Thermodyn..

[20] N’Guimbi J., Berro C., Mokbel I., Rauzy E., Jose J. (1999). Experimental vapour pressures of 13 secondary and tertiary alcohols—Correlation and prediction by a group contribution method. Fluid Phase Equilib..

[21] Štejfa V., Lyshchuk H., Babková K., Krupička M., Ludík J., Fulem M., Červinka C., Růžička K. (2024). Hierarchy of hydrogen bonding among constitutional isomers of hexanol. J. Mol. Liq..

[22] Pokorný V., Štejfa V., Klajmon M., Fulem M., Růžička K. (2021). Vapor Pressures and Thermophysical Properties of 1-Heptanol, 1-Octanol, 1-Nonanol, and 1-Decanol: Data Reconciliation and PC-SAFT Modeling. J. Chem. Eng. Data.

[23] Koutek B., Mahnel T., Šimáček P., Fulem M., Růžička K. (2017). Extracting Vapor Pressure Data from GLC Retention Times. Part 1: Analysis of Single Reference Approach. J. Chem. Eng. Data.

[24] Koutek B., Pokorný V., Mahnel T., Štejfa V., Řehák K., Fulem M., Růžička K. (2022). Estimating Vapor Pressure Data from Gas–Liquid Chromatography Retention Times: Analysis of Multiple Reference Approaches, Review of Prior Applications, and Outlook. J. Chem. Eng. Data.

[25] Herington E.F.G., Desty D.H., Harbourn C.L.A. (1957). Theoretical fundamentals of gas chromatography. Vapour Phase Chromatography, Proceedings of the Symposium Sponsored by the Hydrocarbon Research Group of the Institute of Petroleum, London, UK, 30 May–1 June 1956.

[26] Markovnik V.S., Sachek A.I., Peshchenko A.D., Shvaro O.V., Andreevskii D.N. (1978). Temperature dependence of the vapor pressure of some isostructural aliphatic alcohols. Termodinam. Organ. Soedin. (Gor’kii).

[27] Ambrose D., Ghiassee N.B. (1990). Vapour pressures, critical temperatures, and critical pressures of benzyl alcohol, octan-2-ol, and 2-ethylhexan-1-ol. J. Chem. Thermodyn..

[28] Čenský M., Roháč V., Růžička K., Fulem M., Aim K. (2010). Vapor pressure of selected aliphatic alcohols by ebulliometry. Part 1. Fluid Phase Equilib..

[29] Čenský M., Vrbka P., Růžička K., Fulem M. (2010). Vapor pressure of selected aliphatic alcohols by ebulliometry. Part 2. Fluid Phase Equilib..

[30] Goldberg R.N., Weir R.D. (1992). Conversion of temperatures and thermodynamic properties to the basis of the international temperature scale of 1990. Pure Appl. Chem..

[31] Thomas L.H., Meatyard R., Smith H., Davies G.H. (1979). Vapor pressures and molar entropies of vaporization of monohydric alcohols. J. Chem. Eng. Data.

[32] Kulikov D., Verevkin S.P., Heintz A. (2001). Determination of vapor pressures and vaporization enthalpies of the aliphatic branched C-5 and C-6 alcohols. J. Chem. Eng. Data.

[33] Hovorka F., Lankelma H.P., Stanford S.C. (1938). Thermodynamic Properties of the Hexyl Alcohols. II. Hexanols-1, -2, -3 and 2-Methylpentanol-1 and -4. J. Am. Chem. Soc..

[34] Hovorka F., Lankelma H.P., Naujoks C.K. (1933). Thermodynamic Properties of 2-Methylpentanol-2. J. Am. Chem. Soc..

[35] Hovorka F., Lankelma H.P., Axelrod A.E. (1940). Thermodynamic Properties of the Hexyl Alcohols. III. 2-Methylpentanol-3 and 3-Methylpentanol-3. J. Am. Chem. Soc..

[36] Hovorka F., Lankelma H.P., Bishop J.W. (1941). Thermodynamic Properties of the Hexyl Alcohols. VI. 2,3-Dimethylbutanol-2 and 3-Methylpentanol-21. J. Am. Chem. Soc..

[37] Hovorka F., Lankelma H.P., Schneider I. (1940). Thermodynamic Properties of the Hexyl Alcohols. IV. 3-Methylpentanol-1 and 2-Methylpentanol-5. J. Am. Chem. Soc..

[38] Hovorka F., Lankelma H.P., Smith W.R. (1940). Thermodynamic Properties of the Hexyl Alcohols. V. 2,2-Dimethylbutanol-1 and 2-Ethylbutanol-1. J. Am. Chem. Soc..

[39] N’Guimbi J., Kasehgari H., Mokbel I., Jose J. (1992). Tensions de vapeur d’alcools primaires dans le domaine 0.3 Pa à 1.5 kPa. Thermochim. Acta.

[40] Loginova M.A., Frolov A.F., Bolshako L., Simanov N.A., Obedkova L.V. (1974). Liquid-Vapor-Equilibrium in Systems of Ethylisopropylketone Synthesis. Zh. Prikl. Khim..

[41] Ambrose D., Neindre B., Vodar B. (1975). VaporPressures. Experimental Thermodynamics Volume II.

[42] Zaitsau D.H., Pimerzin A.A., Verevkin S.P. (2019). Fatty acids methyl esters: Complementary measurements and comprehensive analysis of vaporization thermodynamics. J. Chem. Thermodyn..

[43] Majer V., Svoboda V., Uchytilová V., Finke M. (1985). Enthalpies of vaporization of aliphatic C5 and C6 alcohols. Fluid Phase Equilib..

[44] Růžička K., Majer V. (1996). Simple and controlled extrapolation of vapor pressures toward the triple point. AIChE J..

[45] Růžička K., Majer V. (1994). Simultaneous treatment of vapor pressures and related thermal data between the triple and normal boiling temperatures for *n*-alkanes C_5_–C_20_. J. Phys. Chem. Ref. Data.

[46] Tsonopoulos C. (1974). Empirical correlation of second virial coefficients. AIChE J..

[47] Gude M., Teja A.S. (1995). Vapor-Liquid Critical Properties of Elements and Compounds. 4. Aliphatic Alkanols. J. Chem. Eng. Data.

[48] Nannoolal Y., Rarey J., Ramjugernath D. (2007). Estimation of pure component properties: Part 2. Estimation of critical property data by group contribution. Fluid Phase Equilib..

[49] (2019). Artist Software.

[50] Groom C.R., Bruno I.J., Lightfoot M.P., Ward S.C. (2016). The Cambridge Structural Database. Acta Crystallogr. Sect. B.

[51] Kolská Z., Růžička V., Gani R. (2005). Estimation of the Enthalpy of Vaporization and the Entropy of Vaporization for Pure Organic Compounds at 298.15 K and at Normal Boiling Temperature by a Group Contribution Method. Ind. Eng. Chem. Res..

[52] Majer V., Svoboda V. (1985). International Union of Pure and Applied Chemistry Chemical Data Series, No. 32. Enthalpies of Vaporization of Organic Compounds: Critical Review and Data Compilation.

[53] Benson S.W. (1996). Some Observations on the Structures of Liquid Alcohols and Their Heats of Vaporization. J. Am. Chem. Soc..

[54] Serra P.B.P., Růžička K., Fulem M., Vlk O., Krakovský I. (2016). Calorimetric and FTIR study of selected aliphatic heptanols. Fluid Phase Equilib..

[55] Serra P.B.P., Krakovský I., Fulem M., Růžička K. (2018). Calorimetric and FTIR study of selected aliphatic octanols. J. Therm. Anal. Calorim..

[56] Zábranský M., Růžička V. (2004). Estimation of the heat capacities of organic liquids as a function of temperature using group additivity: An amendment. J. Phys. Chem. Ref. Data.

[57] Kolská Z., Kukal J., Zábranský M., Růžička V. (2008). Estimation of the Heat Capacity of Organic Liquids as a Function of Temperature by a Three-Level Group Contribution Method. Ind. Eng. Chem. Res..

[58] Zábranský M., Růžička V., Majer V., Domalski E.S. (1996). Heat Capacity of Liquids. Critical Review and Recommended Values.

[59] Zábranský M., Růžička V., Domalski E.S. (2001). Heat Capacity of Liquids: Critical Review and Recommended Values. Supplement I. J. Phys. Chem. Ref. Data.

[60] Zábranský M., Kolská Z., Růžička V., Domalski E.S. (2010). Heat Capacity of Liquids: Critical Review and Recommended Values. Supplement II. J. Phys. Chem. Ref. Data.

[61] Hamilton D.J. (1980). Gas chromatographic measurement of volatility of herbicide esters. J. Chromatogr. A.

[62] Bidleman T.F. (1984). Estimation of Vapor-Pressures for Nonpolar Organic-Compounds by Capillary Gas-Chromatography. Anal. Chem..

[63] Fischer R., Ballschmiter K. (1989). Relationship between experimentally determined vapour pressures and retention times of PCB on a 50% *n*-octyl-methylpolysiloxane stationary phase. Fresenius’ Z. Anal. Chem..

[64] Hinckley D.A., Bidleman T.F., Foreman W.T., Tuschall J.R. (1990). Determination of Vapor-Pressures for Nonpolar and Semipolar Organic-Compounds from Gas-Chromatographic Retention Data. J. Chem. Eng. Data.

[65] Letcher T.M., Naicker P.K. (2004). Determination of vapor pressures using gas chromatography. J. Chromatogr. A.

[66] Moller B., Rarey J., Ramjugernath D. (2008). Estimation of the vapour pressure of non-electrolyte organic compounds via group contributions and group interactions. J. Mol. Liq..

[67] Nannoolal Y., Rarey J., Ramjugernath D., Cordes W. (2004). Estimation of pure component properties: Part 1. Estimation of the normal boiling point of non-electrolyte organic compounds via group contributions and group interactions. Fluid Phase Equilib..

[68] Meija J., Coplen T.B., Berglund M., Brand W.A., Bièvre P.D., Gröning M., Holden N.E., Irrgeher J., Loss R.D., Walczyk T. (2016). Atomic weights of the elements 2013 (IUPAC Technical Report). Pure Appl. Chem..

[69] Newell D.B., Cabiati F., Fischer J., Fujii K., Karshenboim S.G., Margolis H.S., de Mirandés E., Mohr P.J., Nez F., Pachucki K. (2018). The CODATA 2017 values of *h*,*e*,*k*, and *N*_A_ for the revision of the SI. Metrologia.

[70] Fulem M., Růžička K., Morávek P., Pangrác J., Hulicius E., Kozyrkin B., Shatunov V. (2010). Vapor Pressure of Selected Organic Iodides. J. Chem. Eng. Data.

[71] Pfaendtner J., Yu X., Broadbelt L.J. (2007). The 1-D hindered rotor approximation. Theor. Chem. Acc..

[72] Grimme S., Antony J., Ehrlich S., Krieg H. (2010). A consistent and accurate ab initio parametrization of density functional dispersion correction (DFT-D) for the 94 elements H-Pu. J. Chem. Phys..

[73] Frisch M.J., Trucks G.W., Schlegel H.B., Scuseria G.E., Robb M.A., Cheeseman J.R., Scalmani G., Barone V., Petersson G.A., Nakatsuji H. (2016). Gaussian 16, Revision B.01.

[74] Štejfa V., Fulem M., Růžička K. (2019). First-principles calculation of ideal-gas thermodynamic properties of long-chain molecules by R1SM approach—Application to n-alkanes. J. Chem. Phys..

[75] Marston C.C., Balint-Kurti G.G. (1989). The Fourier grid Hamiltonian method for bound state eigenvalues and eigenfunctions. J. Chem. Phys..

[76] King M.B., Al-Najjar H. (1974). Method for correlating and extending vapor pressure data to lower temperatures using thermal data. Vapor pressure equations for some *n*-alkanes at temperatures below the normal boiling point. Chem. Eng. Sci..

[77] Cox E.R. (1936). Hydrocarbon vapor pressures. Ind. Eng. Chem..

[78] Rodgers T.F.M., Okeme J.O., Parnis J.M., Girdhari K., Bidleman T.F., Wan Y., Jantunen L.M., Diamond M.L. (2021). Novel Bayesian Method to Derive Final Adjusted Values of Physicochemical Properties: Application to 74 Compounds. Environ. Sci. Tech..

[79] Delle Site A. (1997). The vapor pressure of environmentally significant organic chemicals: A review of methods and data at ambient temperature. J. Phys. Chem. Ref. Data.

[80] Orbey H., Sandler S.I. (1991). Relative Measurements of Activity-Coefficients at Infinite Dilution by Gas-Chromatography. Ind. Eng. Chem. Res..

