# Supplementary material for: Vapor Pressure of Selected Aliphatic Hexanols by Static and Indirect Chromatographic Methods
Source: Molecules. 2025 Nov 4;30(21):4287. doi: 10.3390/molecules30214287 (PMC12608578; doi:10.3390/molecules30214287)
Supplement: Supplementary file 1 [file molecules-30-04287-s001.zip › molecules-3954578-supplementary.pdf]

molecules

Supplementary Materials

Vapor Pressure of Selected Aliphatic Hexanols by Static and Indirect Chromatographic Methods

Vapor Pressure of Selected Aliphatic Hexanols by Static and Indirect Chromatographic

Vojtěch Štejfa[1,*], Pavel Šimáček[2], Bohumír Koutek[3], Michal Fulem[1], and Květoslav Růžička[1,*]

1 Department of Physical Chemistry, University of Chemistry and Technology, Prague, Technická 5, CZ-166 28 Prague 6, Czech Republic; stejfav@vscht.cz (V.Š.); fulemm@vscht.cz (M.F.); bkoutek@seznam.cz (B.K.) ; ruzickak@vscht.cz (K.R.)
2 Department of Sustainable Fuels and Green Chemistry, University of Chemistry and Technology, Prague, Technická 5, CZ-166 28 Prague 6, Czech Republic; simacekp@vscht.cz (P.Š)

* Correspondence: ruzickak@vscht.cz , stejfav@vscht.cz

Supplementary materials contain the following:

1) Table S1 with structures and abbreviations of hexanols of this work
2) Section S1 containing experimental values of vapor pressures and sublimation pressures of eight aliphatic hexanols studied in this work (Table S2).
3) Section S2. Properties in the state of ideal gas (containing Table S3, Table S4 and Table S5)
4) Section S3. Description of the SimCor method (containing Table S6)
5) Section S4. Recommended Vaporization Enthalpies (containing Table S7)
6) Section S5. Comparison of experimental and estimated vapor pressures (containing Figure S1)
7) Section S6. Extrapolation of vapor pressures using GLC-ACRT methodology (containing Table S8 and S9)
8) References

 https://doi.org/10.3390/molecules30214287

The abbreviations that are used for compounds of this study throughout this Supporting Material are given in Table S1.

Table S1. List of Abbreviations used for compounds of this study.

Compound	Abbreviation		CAS RN
(±)-3-hexanol	3H	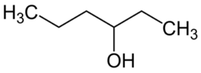	623-37-0
2-methyl-2-pentanol	2M2P	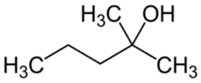	590-36-3
(±)-2-methyl-3-pentanol	2M3P	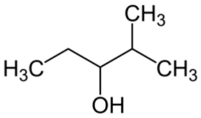	565-67-3
(±)-3-methyl-2-pentanol[e]	3M2P	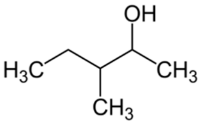	565-60-6
3-methyl-3-pentanol	3M3P	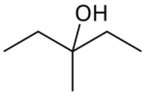	77-74-7
2,2-dimethyl-1-butanol	22M1B	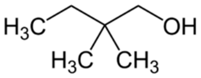	1185-33-7
2,3-dimethyl-2-butanol	23M2B	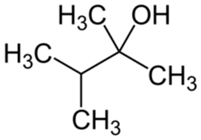	594-60-5
(±)-3,3-dimethyl-2-butanol	33M2B	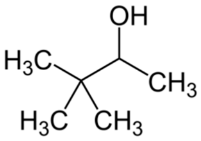	464-07-3

Section S1. Vapor and sublimation pressures

This section contains Table S2 with experimental values of vapor pressures and sublimation pressures of eight aliphatic hexanols studied in this work using apparatus STAT6 described in Section 4.2. Those data are graphically compared in Figure 1 in the main article.

Table S2 Vapor pressures of studied hexanols measured by STAT6 apparatus.[a]

3H (liquid)						2M2P(liquid)					
T / K	p / Pa	T / K	p / Pa	T / K	p / Pa	T / K	p / Pa	T / K	p / Pa	T / K	p / Pa
238.28	0.706	263.23	14.38	288.20	159.61	238.30	2.123	263.24	36.39	288.20	351.96
238.29	0.712	263.23	14.37	288.20	159.51	238.31	2.125	263.24	36.38	288.20	351.94
238.30	0.713	263.23	14.37	288.20	159.59	238.31	2.126	263.24	36.40	288.20	351.94
243.27	1.362	268.21	24.26	293.18	242.70	243.26	3.932	268.22	59.62	293.18	522.67
243.27	1.363	268.21	24.28	293.19	242.82	243.26	3.933	268.22	59.64	293.18	522.70
243.27	1.365	268.21	24.28	293.19	242.82	243.26	3.934	268.22	59.64	293.18	522.67
248.27	2.558	273.20	40.10	298.17	362.39	248.28	7.152	273.20	95.60	298.18	763.66
248.27	2.562	273.20	40.08	298.17	362.38	248.29	7.161	273.20	95.60	298.18	763.71
248.27	2.564	273.20	40.09	298.17	362.42	248.29	7.158	273.20	95.59	298.18	763.70
253.26	4.675	278.22	65.01	303.15	531.95	253.29	12.64	278.22	150.79	303.16	1096.6
253.26	4.676	278.22	64.97	303.15	531.76	253.29	12.65	278.22	150.78	303.16	1096.6
253.26	4.677	278.22	64.97	303.15	531.59	253.29	12.65	278.22	150.79	303.16	1096.6
258.25	8.300	283.21	102.81	308.15	767.99	258.26	21.70	283.21	232.66		
258.25	8.302	283.21	102.78	308.15	767.75	258.26	21.70	283.21	232.66		
258.25	8.301	283.21	102.77	308.15	767.92	258.26	21.70	283.21	232.64		

2M3P (liquid)						3M3P (liquid)					
T / K	p / Pa	T / K	p / Pa	T / K	p / Pa	T / K	p / Pa	T / K	p / Pa	T / K	p / Pa
233.29	0.858	258.26	17.68	283.19	195.14	238.24	0.931	263.18	17.61	288.13	185.33
233.30	0.860	258.26	17.68	288.17	296.13	238.24	0.931	263.18	17.61	293.12	279.57
233.37	0.865	263.23	29.89	288.17	296.15	238.25	0.932	268.14	29.34	293.12	279.31
238.30	1.665	263.24	29.92	288.17	296.22	243.29	1.776	268.14	29.36	293.12	279.32
238.30	1.663	263.24	29.90	293.17	442.09	243.30	1.777	268.20	29.53	298.11	413.86
238.31	1.665	268.22	49.39	293.17	442.05	243.30	1.779	273.14	48.00	298.11	413.85
243.29	3.125	268.22	49.39	293.17	441.99	248.30	3.289	273.14	48.06	298.11	413.82
243.29	3.124	268.22	49.39	298.16	647.42	248.30	3.290	273.14	48.06	303.09	602.11
243.29	3.131	273.21	79.80	298.16	647.19	248.30	3.289	278.13	76.87	303.09	602.13
248.27	5.709	273.21	79.78	298.16	647.19	253.24	5.887	278.13	76.87	303.09	602.03
248.27	5.707	273.21	79.78	303.14	931.56	253.25	5.901	278.13	76.87	308.09	862.00
248.28	5.715	278.20	126.14	303.14	931.64	253.25	5.889	283.13	120.58	308.09	861.98
253.27	10.19	278.20	126.11	303.14	931.73	258.25	10.37	283.13	120.54	308.09	862.33
253.27	10.18	278.20	126.10	308.15	1321.8	258.26	10.37	283.13	120.57		
253.27	10.18	283.19	195.19	308.15	1321.8	258.26	10.37	288.12	185.20		
258.25	17.66	283.19	195.17	308.15	1322.4	263.18	17.60	288.12	185.20		

3M3P (crystal I)		3M3P (liquid)								22M1B (cr I)	
T / K	p / Pa	T / K	p / Pa	T / K	p / Pa	T / K	p / Pa	T / K	p / Pa	T / K	p / Pa
233.31	0.924	233.30	1.341	248.34	8.576	268.30	70.55	288.13	401.74	238.80	0.911
233.31	0.923	233.31	1.338	253.33	15.07	273.29	112.61	293.12	593.76	238.80	0.910
233.38	0.928	233.32	1.343	253.33	15.07	273.29	112.62	293.12	593.63	238.83	0.911
238.29	1.954	238.28	2.540	253.33	15.07	273.30	112.63	293.12	594.03	243.27	1.620
238.29	1.954	238.29	2.541	258.30	25.78	278.21	175.11	298.12	862.69	243.27	1.621
238.31	1.956	238.29	2.540	258.30	25.78	278.21	175.05	298.12	862.67	243.27	1.620
243.27	4.030	238.29	2.542	258.30	25.78	278.21	175.02	298.12	862.89	248.27	3.025
243.27	4.026	243.27	4.707	263.28	43.08	283.18	267.96	303.10	1231.2	248.27	3.025
243.27	4.026	243.27	4.707	263.28	43.06	283.18	267.94	303.11	1231.8	248.27	3.024
248.36	8.222	243.28	4.711	263.29	43.10	283.18	267.94	303.11	1231.8		
248.36	8.207	248.34	8.581	268.29	70.44	288.13	401.74				
248.37	8.244	248.34	8.583	268.29	70.46	288.13	401.72				

22M1B (liquid)				23M2B (cr I)		23M2B (liquid)				33M2B (cr II)	
T / K	p / Pa	T / K	p / Pa	T / K	p / Pa	T / K	p / Pa	T / K	p / Pa	T / K	p / Pa
253.26	5.459	283.20	109.81	238.30	2.532	253.28	20.71	278.22	229.29	233.73	1.223
253.26	5.457	283.20	109.81	238.30	2.525	253.28	20.70	278.22	229.27	233.74	1.232
253.26	5.453	283.20	109.81	238.30	2.521	253.28	20.72	278.22	229.29	233.74	1.228
258.26	9.538	288.18	168.51	243.26	4.980	258.26	35.03	283.20	347.95	233.75	1.226
258.26	9.544	288.18	168.52	243.26	4.967	258.26	35.02	283.20	347.95	238.34	2.327
258.26	9.545	288.18	168.51	243.27	4.966	258.26	35.02	283.20	348.00	238.34	2.320
263.23	16.23	293.17	253.83	248.30	9.582	263.25	57.88	288.19	518.71	238.34	2.318
263.24	16.25	293.18	253.96	248.30	9.599	263.25	57.87	288.19	518.67	243.31	4.492
263.24	16.24	293.18	253.97	248.30	9.603	263.25	57.89	288.19	518.68	243.32	4.509
268.21	26.99	298.16	375.89	253.27	17.99	268.22	93.29	293.17	759.12	243.32	4.494
268.22	27.02	298.16	375.93	253.28	17.98	268.22	93.33	293.17	759.08	248.33	8.554
268.22	27.03	298.16	375.84	253.28	17.98	268.22	93.32	293.17	759.08	248.33	8.538
273.21	43.98	303.15	547.01	258.26	32.76	268.22	93.35	298.16	1092.9	253.27	17.99
273.21	44.00	303.15	546.56	258.26	32.78	268.22	93.39	298.16	1092.8	253.31	15.71
273.21	44.01	303.15	546.92	258.26	32.83	268.23	93.39			253.32	15.80
278.21	70.26	308.15	783.79			273.20	147.39			253.32	15.76
278.21	70.26	308.15	783.32			273.20	147.39				
278.21	70.26	308.15	783.69			273.21	147.52				

33M2B (cr I)											
T / K	p / Pa	T / K	p / Pa	T / K	p / Pa	T / K	p / Pa	T / K	p / Pa	T / K	p / Pa
248.33	8.753	253.33	15.88	258.27	27.77	268.25	81.15	273.23	134.00	278.22	217.52
248.34	8.782	253.33	15.85	263.26	47.98	268.25	81.12	273.23	134.00		
248.34	8.798	258.27	27.82	263.26	47.95	268.25	81.09	278.22	217.57		
253.32	15.82	258.27	27.79	263.26	47.94	273.23	134.15	278.22	217.45		

33M2B (liquid)											
T / K	p / Pa	T / K	p / Pa	T / K	p / Pa	T / K	p / Pa	T / K	p / Pa	T / K	p / Pa
273.24	142.48	278.17	217.98	283.17	329.41	288.15	488.56	293.14	712.44	298.13	1021.6
273.24	142.47	278.18	218.19	283.17	329.50	288.16	488.74	293.14	712.26	298.13	1021.3
273.24	142.48	278.18	218.14	283.17	329.38	288.16	488.78	293.14	712.77		

[a] The standard uncertainty in the sample temperature measurements is $u(T)$ = 0.01 K, and the combined expanded uncertainty (0.95 level of confidence, k = 2) in the vapor pressure measurements is $U_c(p) = 0.005p + 0.05$ Pa.
[b]Values are reported with one digit more than is justified by the experimental uncertainty to avoid round-off errors in calculations based on these results.

Section S2. Properties in the state of ideal gas

This section contains numerical values related to ideal-gas properties. Methodology is described in Section 4.3, and results are discussed in Section 2.3.

Table S3 lists stable conformers of studied compounds, Table S4 contains parameters of symmetrical and terminal top rotations in the studied hexanols, and Table S5 contains standard molar thermodynamic functions of the studied hexanols in the ideal gaseous state at $p = 10^5$ Pa.

Table S3 Stable Conformers Treated in the R1TM as Optimized at the B3LYP/6-311+G(2df,p) Level of Theory and Their Relative Energies, Symmetries, Dipole Moments μ, and Products of Inertia I_{ABC}^{calc} and excluded frequencies

3H

φ_1 = 1-2-3-4; φ_2 = 2-3-7-21;
φ_3 = 2-3-4-5; φ_3 = 3-4-5-6

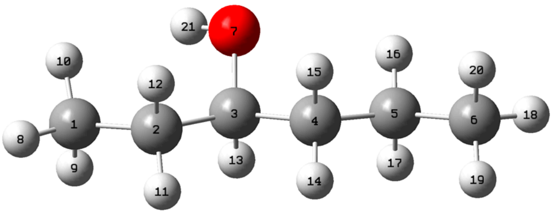

Conformer	Label [a]	$\Delta_r H_{ij}^0$ / kJ mol^{-1}	μ / D	Symmetry	I_{ABC}^{calc} / 10^{-135} kg·m^2	Excluded frequencies [b]	φ_1	φ_2	φ_3	φ_4
4	t(t)gt	2.65	1.675	C_1	96.667	5 7 8	−177.5	−179.8	67.3	174.8
5	t(t)gg	5.64	1.706	C_1	86.005	5 6 7	−178.5	−179.5	62.0	59.4
6	t(t)gd'	11.31	1.698	C_1	88.321	6 7 8	−179.3	−179.9	62.3	−97.2
7	t(t)g't	3.77	1.437	C_1	82.913	5 6 7	−171.6	−174.2	−58.9	−173.1
8	t(t)g'g'	8.83	1.513	C_1	69.634	5 6 7	−170.8	−178.7	−52.2	−65.7
9	t(t)g'd	11.30	1.506	C_1	78.733	5 6 7	−172.3	−175.1	−59.4	93.0
10	t(t)dg'	12.28	1.739	C_1	86.917	4 6 7	−172.7	−175.3	93.0	−63.9
23	t(g')tt	0.00	1.619	C_1	80.663	5 6 7	−178.9	−65.6	174.6	179.2
24	t(g')tg	6.48	1.735	C_1	70.870	5 6 7	−179.2	−62.4	169.0	75.7
25	t(g')tg'	2.27	1.524	C_1	77.548	5 6 7	−178.3	−64.7	179.2	−64.3
37	g(t)gt	4.90	1.655	C_1	89.685	5 6 7	56.2	176.3	59.7	174.6
39	g(t)gd'	13.23	1.704	C_1	72.791	5 7 8	60.1	178.4	64.0	−90.4
52	g(g')tt	3.39	1.546	C_1	88.539	5 6 8	59.1	−61.0	171.2	178.9
53	g(g')tg	9.58	1.618	C_1	70.112	4 6 7	58.7	−57.7	163.0	71.2
54	g(g')tg'	6.16	1.444	C_1	73.388	5 6 7	59.5	−60.6	175.0	−65.8
56	g(g')gg	8.17	1.553	C_1	75.434	4 6 7	56.8	−60.7	56.5	60.6
60	g(g')d'd'	22.02	1.709	C_1	65.001	4 6 8	60.1	−56.2	−87.7	−88.1
65	g'(t)g'g'	10.10	1.520	C_1	69.458	4 6 7	−59.9	178.1	−51.1	−65.8
66	g'(t)g'd	13.04	1.510	C_1	73.842	6 7 8	−63.9	−178.3	−60.2	92.9
79	g'(g')tt	2.21	1.642	C_1	103.053	5 6 8	−67.4	−56.6	176.5	179.6
80	g'(g')tg	8.71	1.757	C_1	78.618	3 5 8	−69.5	−54.7	168.9	74.3
81	g'(g')tg'	4.70	1.552	C_1	85.068	6 7 8	−66.5	−55.9	−178.7	−63.7
82	g'(g')g't	4.90	1.653	C_1	91.118	6 7 8	−59.6	−51.7	−56.5	−173.9
85	g'(g')dt	11.63	1.698	C_1	102.096	6 7 8	−60.9	−51.1	96.0	−178.7

86	g'(g')dg	14.69	1.662	C_1	87.923	5 6 7	−61.6	−51.4	98.4	72.6
88	g(t)g't	15.59	1.473	C_1	80.757	5 6 7	79.6	−173.5	−62.5	−167.2
89	d(t)g'g'	20.12	1.530	C_1	59.628	5 6 7	82.9	−177.9	−56.4	−64.0
90	d(t)g'd	24.17	1.540	C_1	65.732	5 6 8	83.5	−174.1	−60.5	96.1
97	d'(t)gt	11.79	1.667	C_1	104.382	5 6 7	−93.3	174.6	61.0	174.0
98	d'(t)gg	14.45	1.691	C_1	81.167	5 6 7	−91.5	174.4	61.8	62.8
99	d'(t)gd'	20.68	1.696	C_1	80.810	5 6 7	−91.4	174.8	62.4	−92.2

2M2P

φ_1 = 3-2-8-21; φ_2 = 8-2-3-4; φ_3 = 2-3-4-5

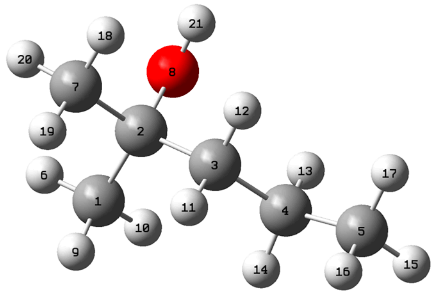

7	(g)gt	0.00	1.485	C_1	69.868	4 5 6 7	63.7	59.1	−172.1
9	(g)gg'	5.78	1.556	C_1	54.595	3 4 5 6	58.5	66.7	−65.8
12	(g')g'd'	7.98	1.543	C_1	60.069	3 4 7 8	−61.7	−59.8	−95.6
25	(g)tt	0.73	1.702	C_1	72.538	4 5 6 7	59.1	177.3	−179.9
27	(g)td	9.83	1.676	C_1	62.137	4 5 6 8	60.7	−178.1	93.3
30	(g)n'g'	12.46	1.687	C_1	56.808	3 5 6 8	70.1	−43.7	−68.2
33	(g)tg	10.78	1.693	C_1	58.609	2 3 6 7	60.5	161.7	65.3

2M3P

φ_1 = 11-2-3-4; φ_2 = 2-3-8-21; φ_3 = 2-3-4-5

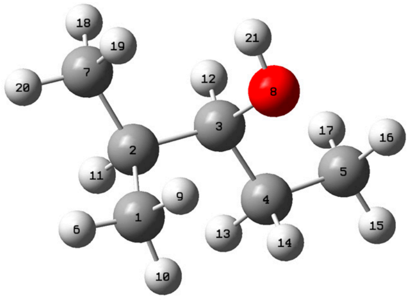

1	g(t)t	1.20	1.559	C_1	68.557	3 4 5 7	63.7	−170.6	−177.2
2	g(t)d	10.43	1.558	C_1	68.630	3 4 6 8	65.3	−176.6	94.5
3	g(t)g'	2.51	1.493	C_1	60.975	4 5 6 7	65.4	−175.2	−57.1
16	g'(g')t	0.00	1.539	C_1	63.876	4 5 6 7	−59.2	−68.0	169.6
17	g'(g')g	2.22	1.560	C_1	63.342	3 6 8 9	−61.9	−64.7	57.7
18	g'(g')d'	13.42	1.588	C_1	56.421	5 6 7 9	−59.1	−65.1	−86.6
25	t(g')t	1.21	1.563	C_1	67.032	4 5 6 7	174.0	−60.4	171.8
26	t(g')g'	12.64	1.584	C_1	57.824	2 3 6 7	−174.2	−54.9	−68.7
27	t(g')d	11.89	1.566	C_1	65.947	3 4 7 8	−179.7	−53.0	94.1
29	n'(g)g'	13.76	1.759	C_1	59.819	3 4 5 9	−32.6	67.9	−61.6
31	t(t)g	11.06	1.568	C_1	62.850	3 5 6 7	161.9	174.1	64.2

3M3P

φ_1 = 1-2-3-4; φ_2 = 2-3-6-18; φ_3 = 2-3-4-5

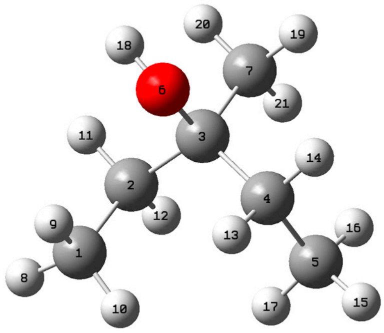

3	t(t)g'	1.13	1.505	C_1	56.181	3 5 6 7	−177.2	−174.4	−57.2
4	t(g)t	0.56	1.550	C_s	53.353	4 5 6 7	177.8	59.8	−177.8
15	g(g)d'	10.93	1.699	C_1	54.644	3 5 7 8	57.5	60.8	−88.1
17	g(g')g	0.00	1.518	C_1	54.691	3 4 6 7	54.6	−64.2	57.3
22	g'(g)t	1.29	1.630	C_1	57.790	3 5 6 8	−65.3	55.6	−176.8
27	g'(g')d	10.83	1.611	C_1	56.480	3 5 6 7	−60.5	−54.1	87.3

22M1B

φ_1 = 3-1-2-4; φ_2 = 1-2-4-5; φ_3 = 2-4-5-6

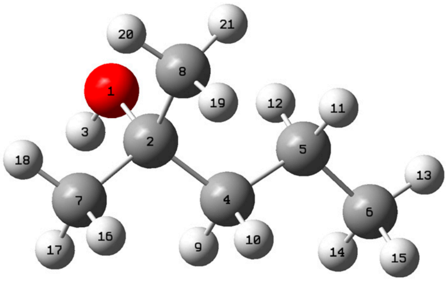

7	(g)tt	0.73	1.702	C_1	72.539	4 5 6 7	59.1	177.3	−179.9
8	(g)tg	10.79	1.693	C_1	58.594	2 3 6 7	60.3	161.7	65.2
10	(g)gt	0.00	1.485	C_1	69.868	4 5 6 7	63.7	59.1	−172.1
11	(g)gd	7.98	1.542	C_1	60.058	3 4 7 8	62.0	59.8	95.6
16	(g)gg'	5.78	1.556	C_1	54.590	3 4 5 6	58.8	66.7	−65.8

23M2B

φ_1 = 3-1-2-4; φ_2 = 1-2-4-9

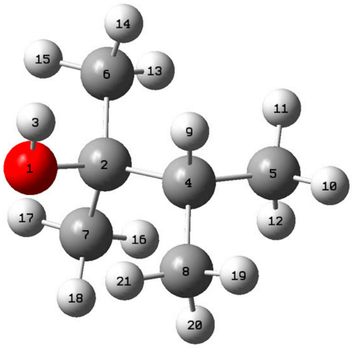

3	(g)t	1.73	1.623	C_1	46.421	2 3 4 5 7	70.5	−179.2
5	(g)g'	0.00	1.594	C_1	47.359	2 3 4 5 7	63.3	−58.4

	33M2B					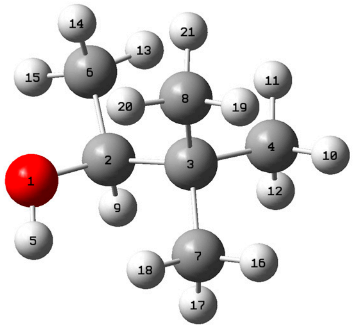			
	φ_1 = 3-2-1-5								
3	(g')	0.00	1.572	C_1	47.017	1 2 3 4 5 7	−66.2		
	(2*R*,3*R*)-3M2P					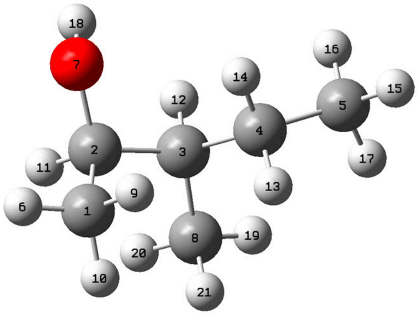			
	φ_1 = 18-7-2-3; φ_2 = 7-2-3-4; φ_3 = 2-3-4-5								
1	(g)g't	0.00	1.667	C_1	66.561	3 5 6 7	64.5	−64.0	−164.5
5	(g')g'g	6.19	1.675	C_1	57.647	3 5 6 7	−71.4	−73.7	67.6
11	(g')g'g'	0.62	1.639	C_1	65.230	3 4 6 7	−72.2	−58.6	−61.9
14	(t)tt	1.56	1.590	C_1	70.950	4 5 6 7	179.0	176.4	−179.1
17	(t)tg	3.01	1.538	C_1	63.089	3 4 5 6	177.8	174.0	59.3
19	(t)td'	7.24	1.557	C_1	69.335	3 4 6 8	−178.9	177.2	−95.6
23	(g)gt	0.22	1.541	C_1	69.332	3 4 5 8	57.8	62.4	−162.9
26	(g)gd	10.35	1.611	C_1	60.479	3 5 7 8	57.1	61.7	88.8
29	(g)gg'	4.27	1.594	C_1	60.858	3 4 6 7	53.9	71.0	−62.2
118	(g)o'g'	12.58	1.688	C_1	68.915	3 5 6 8	66.1	−150.9	−59.9
	(2*R*,3*S*)-3M2P					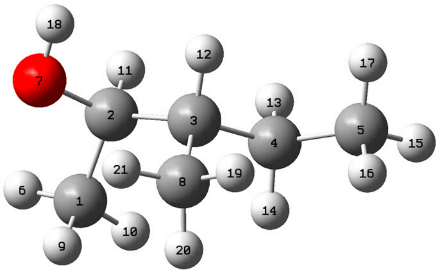			
	φ_1 = 18-7-2-3; φ_2 = 7-2-3-4; φ_3 = 2-3-4-5								
2	(g')g't	0.35	1.580	C_1	71.993	4 5 6 7	−67.9	−57.3	164.7
8	(t)g'g'	2.07	1.474	C_1	61.962	3 4 5 6	−177.7	−51.6	−61.7
10	(t)g'd	5.86	1.654	C_1	66.593	4 5 6 7	179.5	−53.7	89.4
12	(g)tt	0.00	1.655	C_1	68.110	4 5 6 8	57.7	−170.9	167.3
15	(g)tg	1.58	1.705	C_1	66.687	3 4 5 7	58.5	−174.1	58.1
18	(g)o'g'	10.72	1.732	C_1	61.773	3 5 6 7	58.0	−155.1	−65.4
23	(g)gt	1.19	1.558	C_1	67.603	3 4 5 7	63.7	61.4	176.8
26	(g)gd	6.60	1.587	C_1	65.274	3 4 6 8	63.0	62.0	96.4

28	(t)ng	9.91	1.479	C_1	60.549	3 4 7 8	173.3	40.5	59.9
29	(g)gg'	5.38	1.616	C_1	55.354	3 4 5 8	61.1	70.6	−64.2

[a] Labels for dihedral angles are: c = 0–15° (cis), n = 15-45° (narrowed gauche), g = 45–80° (gauche), d = 80–105° (distorted gauche), e = 105–135° (eclipsed), a = 135–160° (anticlinal), and t = 160–180° (trans). Primes denote anticlockwise (negative) rotation. Hydroxyl group rotations are always distinguished by brackets.

[b] Numbers of frequencies identified to correspond to internal rotations treated as 1-DHR.

Table S4 Parameters of Symmetrical and Terminal Top Rotations in the Hexanols

Compound	Top [a]	Internal symmetry number	Reduced moment of inertia [b] I_r / 10^{-47} kg m^2	Fourier expansion parameters [b,c] / J mol^{-1}
3H	hydroxyl 3-7	1	1.319	2611, −635, 842, 206, 664, −2182, −770
	methyl 5-6	3	5.042	6251, 0, 0, 0, 0, −6346, 0, 0, 0, 0, 0, 95, 0
	methyl 1-2	3	5.026	5824, 0, 0, 0, 0, −5891, 0, 0, 0, 0, 0, 67, 0
2M2P	hydroxyl 2-8	1	1.379	2747, 277, 764, −610, 428, −2413, −626
	methyl 2-7	3	5.196	7433, 0, 0, 0, 0, −7638, −155, 0, 0, 0, 0, 207, 0
	methyl 1-2	3	5.158	6768, 0, 0, 0, 0, −6926, −96, 0, 0, 0, 0, 159, 100
	methyl 4-5	3	5.092	6317, 0, 0, 0, 0, −6417, −16, 0, 0, 0, 0, 100, 12
2M3P	hydroxyl 3-8	1	1.356	3174, −569, 1075, −646, 1147, −1959, −1160
	methyl 2-7	3	5.120	6707, 0, 0, 0, 0, −6806, 101, 0, 0, 0, 0, 100, 0
	methyl 1-2	3	5.164	5782, 0, 0, 0, 0, −5850, −259, 0, 0, 0, 0, 68, 138
	methyl 4-5	3	5.104	5771, 0, 0, 0, 0, −5872, 0, 0, 0, 0, 0, 101, 0
3M3P	hydroxyl 3-6	1	1.364	2698, 431, 900, −786, 390, −2341, −679
	methyl 3-7	3	5.165	6981, 0, 0, 0, 0, −7140, −261, 0, 0, 0, 0, 164, 0
	methyl 1-2	3	5.138	5995, 0, 0, 0, 0, −5993, −145
	methyl 4-5	3	5.117	5663, 0, 0, 0, 0, −5663, 0
22M1B	hydroxyl 1-2	1	1.379	2747, 277, −764, −610, −428, −2413, 626
	methyl 2-8	3	5.196	6768, 0, 0, 0, 0, −6926, 95, 0, 0, 0, 0, 159, −100
	methyl 2-7	3	5.158	7433, 0, 0, 0, 0, −7640, 0, 0, 0, 0, 0, 207, 0
	methyl 5-6	3	5.092	6317, 0, 0, 0, 0, −6417, 0, 0, 0, 0, 0, 100, 0
23M2B	hydroxyl 1-2	1	1.371	3149, 59, −1031, −835, −336, −2372, 644
	methyl 2-7	3	5.173	6123, 0, 0, 0, 0, −6339, 0, 0, 0, 0, 0, 216, 0
	methyl 2-6	3	5.159	7600, 0, 0, 0, 0, −7724, 675, 0, 0, 0, 0, 124, −317
	methyl 4-8	3	5.143	6215, 0, 0, 0, 0, −6211, 377, 0, 0, 0, 0, 0, −298
	methyl 4-5	3	5.120	5992, 0, 0, 0, 0, −6180, 273, 0, 0, 0, 0, 189, −104
33M2B	hydroxyl 1-2	1	1.351	2885, 574, 1541, −1507, 681, −1948, −1116
	tert-butyl 2-3	3	63.527	12622, 0, 0, 0, 268, −13750, −1065, 0, 270, 0, 0, 1144, 0
	methyl 2-6	3	5.150	5594, 0, 0, 0, 0, −5832, 0, 0, 0, 0, 0, 237, 0
	methyl 3-8	3	5.163	6679, 0, 0, 0, 0, −6781, −357, 0, 0, 0, 0, 102, 213
	methyl 3-7	3	5.138	7533, 0, 0, 0, 0, −7704, −216, 0, 0, 0, 0, 171, 149
	methyl 3-4	3	5.135	6842, 0, 0, 0, 0, −7069, 219, 0, 0, 0, 0, 228, −125

Compound	Top [a]	Internal symmetry number	Reduced moment of inertia [b] I_r / 10^{-47} kg m^2	Fourier expansion parameters [b,c] / J mol^{-1}
(2*R*,3*R*)-3M2P	hydroxyl 2-7	1	1.368	3265, 232, −868, −1276, 0, −2220, 231
	methyl 1-2	3	5.183	6101, 0, 0, 0, 0, −6226, 0, 0, 0, 0, 0, 125, 0
	methyl 3-8	3	5.142	5380, 0, 0, 0, 0, −5520, −56, 0, 0, 0, 0, 140, 39
	methyl 4-5	3	5.096	5548, 0, 0, 0, 0, −5685, −104, 0, 0, 0, 0, 137, 0
(2*R*,3*S*)-3M2P	hydroxyl 2-7	1	1.351	3240, 242, −836, −1239, 0, −2243, 250
	methyl 1-2	3	5.184	6019, 0, 0, 0, 0, −6147, −114, 0, 0, 0, 0, 130, 0
	methyl 3-8	3	5.166	5665, 0, 0, 0, 0, −5665, 335, 0, 0, 0, 0, 0, −161
	methyl 4-5	3	5.084	5752, 0, 0, 0, 0, −5871, 182, 0, 0, 0, 0, 123, 0

[a] Consult Table S3 for atom numbers.

[b] As calculated for the lowest-energy conformer.

[c] Parameters of the equation $V = V_0 + \sum_i \left(V_i^{\cos} \cos(i\varphi) + V_i^{\sin} \sin(i\varphi) \right)$ listed in the order V_0, $V_1^{\cos}$, $V_1^{\sin}$, $V_2^{\cos}$, $V_2^{\sin}$, etc.·

Table S5 Standard Molar Thermodynamic Functions (in J·K^{-1}·mol^{-1}) of the Hexanols in the Ideal Gaseous State at $p = 10^5$ Pa [a]

	3H (racemic)			2M2P			2M3P (racemic)		
μ [b]	1.603 D			1.583 D			1.548 D		
T / K	$C_{p,\mathrm{m}}^{\mathrm{g0}}$	$S_{\mathrm{m}}^{\mathrm{g0}}$	$H_{\mathrm{m}}^{\mathrm{g0}}/T$	$C_{p,\mathrm{m}}^{\mathrm{g0}}$	$S_{\mathrm{m}}^{\mathrm{g0}}$	$H_{\mathrm{m}}^{\mathrm{g0}}/T$	$C_{p,\mathrm{m}}^{\mathrm{g0}}$	$S_{\mathrm{m}}^{\mathrm{g0}}$	$H_{\mathrm{m}}^{\mathrm{g0}}/T$
100	93.5	298.5	59.6	76.1	290.6	52.7	84.3	293.3	58.7
150	111.9	340.3	74.3	99.4	326.0	64.5	102.6	330.9	70.3
200	126.1	374.4	85.4	120.4	357.5	75.8	120.5	362.9	80.6
210	129.1	380.6	87.5	124.6	363.4	78.1	124.3	368.9	82.6
220	132.2	386.7	89.4	128.8	369.3	80.3	128.1	374.7	84.6
230	135.4	392.6	91.3	133.0	375.1	82.5	131.9	380.5	86.6
240	138.8	398.5	93.3	137.3	380.9	84.7	135.9	386.2	88.5
250	142.2	404.2	95.1	141.6	386.6	86.9	139.9	391.8	90.5
260	145.8	409.8	97.0	146.0	392.2	89.1	144.0	397.4	92.5
270	149.4	415.4	98.9	150.3	397.8	91.2	148.2	402.9	94.5
273.15	150.6	417.1	99.5	151.7	399.6	91.9	149.5	404.6	95.1
280	153.2	420.9	100.8	154.7	403.4	93.4	152.4	408.4	96.5
290	157.0	426.3	102.6	159.1	408.9	95.6	156.6	413.8	98.5
298.15	160.1	430.7	104.2	162.7	413.3	97.4	160.0	418.2	100.1
300	160.8	431.7	104.5	163.5	414.3	97.8	160.8	419.2	100.5
310	164.7	437.1	106.4	167.9	419.8	100.0	165.1	424.5	102.5
320	168.7	442.4	108.3	172.2	425.2	102.2	169.4	429.8	104.5
330	172.7	447.6	110.2	176.6	430.5	104.4	173.6	435.1	106.5
340	176.7	452.8	112.1	180.9	435.9	106.6	177.9	440.4	108.6
350	180.6	458.0	114.0	185.2	441.2	108.7	182.1	445.6	110.6
360	184.6	463.2	115.9	189.5	446.4	110.9	186.3	450.8	112.7
370	188.6	468.3	117.8	193.7	451.7	113.1	190.5	455.9	114.7
380	192.6	473.4	119.7	197.8	456.9	115.3	194.6	461.1	116.8
390	196.5	478.4	121.6	201.9	462.1	117.5	198.7	466.2	118.8
400	200.5	483.4	123.5	206.0	467.3	119.6	202.7	471.3	120.9
500	237.3	532.2	142.7	242.9	517.3	140.7	240.0	520.6	141.1
600	269.1	578.3	161.2	273.8	564.4	160.4	271.2	567.2	160.2
700	296.2	621.9	178.6	299.7	608.6	178.5	297.6	611.0	178.0
$100u_r$ [c]	0.6	0.6	0.7	0.5	0.6	0.7	0.6	0.6	0.7

Table S5. continued

	3M3P			22M1B			23M2B (racemic)		
μ [b]	1.553 D			1.582 D			1.605 D		
T / K	$C_{p,\mathrm{m}}^{\mathrm{g0}}$	$S_{\mathrm{m}}^{\mathrm{g0}}$	$H_{\mathrm{m}}^{\mathrm{g0}}/T$	$C_{p,\mathrm{m}}^{\mathrm{g0}}$	$S_{\mathrm{m}}^{\mathrm{g0}}$	$H_{\mathrm{m}}^{\mathrm{g0}}/T$	$C_{p,\mathrm{m}}^{\mathrm{g0}}$	$S_{\mathrm{m}}^{\mathrm{g0}}$	$H_{\mathrm{m}}^{\mathrm{g0}}/T$
100	77.6	290.3	53.8	76.1	290.6	52.7	76.1	281.1	51.3
150	99.5	326.0	65.5	99.3	325.9	64.4	100.5	316.8	63.7
200	119.5	357.4	76.5	119.8	357.3	75.7	121.7	348.6	75.6
210	123.5	363.4	78.6	123.9	363.3	77.9	125.8	354.6	77.9
220	127.6	369.2	80.8	127.9	369.1	80.1	130.0	360.6	80.2
230	131.6	375.0	82.9	132.1	374.9	82.3	134.1	366.5	82.4
240	135.7	380.6	85.0	136.2	380.6	84.4	138.2	372.3	84.7
250	139.9	386.3	87.1	140.4	386.3	86.6	142.4	378.0	86.9
260	144.0	391.8	89.2	144.6	391.9	88.7	146.6	383.7	89.1
270	148.2	397.3	91.3	148.8	397.4	90.9	150.8	389.3	91.3
273.15	149.6	399.1	92.0	150.1	399.1	91.6	152.1	391.0	92.0
280	152.5	402.8	93.4	153.0	402.9	93.0	155.0	394.8	93.5
290	156.7	408.2	95.6	157.3	408.3	95.2	159.2	400.3	95.7
298.15	160.2	412.6	97.3	160.8	412.7	96.9	162.6	404.8	97.5
300	161.0	413.6	97.7	161.6	413.7	97.3	163.4	405.8	97.9
310	165.2	419.0	99.8	165.9	419.1	99.5	167.6	411.2	100.1
320	169.4	424.3	101.9	170.1	424.4	101.6	171.8	416.6	102.3
330	173.7	429.6	104.0	174.4	429.7	103.7	175.9	422.0	104.4
340	177.9	434.8	106.1	178.6	435.0	105.9	180.1	427.3	106.6
350	182.1	440.0	108.2	182.8	440.2	108.0	184.2	432.6	108.7
360	186.2	445.2	110.3	187.0	445.4	110.2	188.2	437.8	110.9
370	190.3	450.4	112.4	191.2	450.6	112.3	192.3	443.0	113.0
380	194.4	455.5	114.5	195.3	455.8	114.4	196.3	448.2	115.2
390	198.5	460.6	116.6	199.3	460.9	116.6	200.2	453.3	117.3
400	202.4	465.7	118.7	203.3	466.0	118.7	204.1	458.5	119.4
500	239.2	514.9	139.2	240.2	515.4	139.4	239.8	507.9	140.0
600	270.2	561.3	158.6	271.2	562.1	158.8	269.9	554.4	159.2
700	296.5	605.0	176.4	297.5	605.9	176.8	295.5	598.0	176.9
$100u_{\mathrm{r}}$ [c]	0.6	0.6	0.7	0.6	0.6	0.7	0.5	0.5	0.7

Table S5. continued

	33M2B (racemic)			(2*R*,3*R*)-3M2P (enantiopure)			(2*R*,3*S*)-3M2P (enantiopure)		
μ [b]	1.572 D			1.614 D			1.605 D		
T / K	$C_{p,\mathrm{m}}^{\mathrm{g}0}$	$S_{\mathrm{m}}^{\mathrm{g}0}$	$H_{\mathrm{m}}^{\mathrm{g}0}/T$	$C_{p,\mathrm{m}}^{\mathrm{g}0}$	$S_{\mathrm{m}}^{\mathrm{g}0}$	$H_{\mathrm{m}}^{\mathrm{g}0}/T$	$C_{p,\mathrm{m}}^{\mathrm{g}0}$	$S_{\mathrm{m}}^{\mathrm{g}0}$	$H_{\mathrm{m}}^{\mathrm{g}0}/T$
100	70.6	273.4	48.6	81.5	291.8	57.1	82.3	291.1	57.8
150	96.4	307.1	60.4	102.6	329.0	68.9	102.9	328.5	69.5
200	118.6	337.9	72.2	121.2	361.1	79.7	121.6	360.6	80.2
210	122.9	343.8	74.5	124.9	367.1	81.7	125.4	366.7	82.2
220	127.2	349.6	76.8	128.7	373.0	83.8	129.1	372.6	84.3
230	131.4	355.3	79.1	132.5	378.8	85.8	133.0	378.4	86.3
240	135.7	361.0	81.3	136.3	384.5	87.8	136.8	384.1	88.3
250	140.0	366.7	83.6	140.2	390.2	89.8	140.7	389.8	90.4
260	144.3	372.2	85.9	144.1	395.8	91.9	144.7	395.4	92.4
270	148.6	377.8	88.1	148.0	401.3	93.9	148.7	400.9	94.4
273.15	150.0	379.5	88.8	149.3	403.0	94.5	149.9	402.7	95.0
280	152.9	383.2	90.3	152.0	406.7	95.9	152.7	406.4	96.4
290	157.3	388.7	92.6	156.1	412.1	97.9	156.7	411.8	98.4
298.15	160.8	393.1	94.4	159.3	416.5	99.5	160.0	416.2	100.0
300	161.6	394.1	94.8	160.1	417.5	99.9	160.8	417.2	100.4
310	165.9	399.5	97.0	164.1	422.8	101.9	164.9	422.6	102.4
320	170.2	404.8	99.2	168.2	428.1	103.9	168.9	427.9	104.4
330	174.4	410.1	101.5	172.3	433.3	105.9	173.0	433.1	106.5
340	178.7	415.4	103.7	176.3	438.5	107.9	177.1	438.3	108.5
350	182.9	420.6	105.9	180.4	443.7	109.9	181.1	443.5	110.5
360	187.1	425.8	108.1	184.4	448.8	111.9	185.1	448.7	112.5
370	191.2	431.0	110.3	188.4	453.9	114.0	189.1	453.8	114.5
380	195.3	436.2	112.4	192.3	459.0	116.0	193.1	458.9	116.5
390	199.3	441.3	114.6	196.3	464.1	118.0	197.0	464.0	118.6
400	203.3	446.4	116.8	200.2	469.1	120.0	200.9	469.0	120.6
500	239.8	495.8	137.8	236.4	517.7	139.7	237.1	517.8	140.3
600	270.4	542.3	157.4	267.5	563.7	158.5	268.1	563.9	159.1
700	296.1	585.9	175.5	294.1	607.0	176.0	294.5	607.2	176.6
$100u_r$ [c]	0.6	0.5	0.7	0.6	0.6	0.7	0.6	0.6	0.7

[a] Values were calculated combining R1TM approach with B3LYP-D3/6-311+G(2df,p) quantum-chemical calculations. Calculated fundamental frequencies were scaled by a double-linear scaling factor SF(v > 2000 cm^{-1}) = 0.960; SF(v < 2000 cm^{-1}) = 0.9948 − 1.35·10^{-5}v.[1]

[b] Dipole moment at 298.15 in Debye calculated using the R1TM approach.

[c] Standard relative uncertainty estimated according to the scheme presented in ref [1].

Section S3. Description of the SimCor method

This section describes simultaneous correlation of vapor pressures and related thermal properties (SimCor method) in more details than section 4.4.

SimCor was suggested (in a simplified form) by King and Al-Najjar in 1974 [2]. The SimCor is based on exact thermodynamic relationships and the procedure must therefore yield reliable results providing that the input data are of reasonable accuracy. A great advantage of this approach is that a single equation can furnish a description of the temperature dependences of several thermodynamic properties and the SimCor thus also provides a test on the consistency of different experimental data.

A full description of the SimCor Method was presented previously by Růžička and Majer [3,4], however it is repeated here for reader's convenience.

Let us define auxiliary quantities $\Delta H'$ and $\Delta C'$:

$$\Delta H' \equiv \Delta_{\mathrm{cd}}^{\mathrm{g}} H_{\mathrm{m}} / \Delta_{\mathrm{cd}}^{\mathrm{g}} z \tag{S1}$$

$$\Delta C' \equiv \left(\mathrm{d}\Delta H' / \mathrm{d}T\right)_{\mathrm{sat}} = \left[\Delta_{\mathrm{cd}}^{\mathrm{g}} C_{p,\mathrm{m}} - 2\Delta H' \left(\partial \Delta_{\mathrm{cd}}^{\mathrm{g}} z / \partial T\right)_{p} - \frac{p}{RT} \Delta H'^{2} \left(\partial \Delta_{\mathrm{cd}}^{\mathrm{g}} z / \partial p\right)_{T}\right] \Big/ \Delta_{\mathrm{cd}}^{\mathrm{g}} z \tag{S2}$$

where $\Delta_{\mathrm{cd}}^{\mathrm{g}} z$ stands for the difference between the compressibility factors of the coexisting condensed and gas phases, $\Delta_{\mathrm{cd}}^{\mathrm{g}} H_{\mathrm{m}}$ is the vaporization/sublimation enthalpy and $\Delta_{\mathrm{cd}}^{\mathrm{g}} C_{p,\mathrm{m}} = C_{p,\mathrm{m}}^{\mathrm{g}} - C_{p,\mathrm{m}}^{\mathrm{cd}}$ is the difference between isobaric heat capacity of gas and that of condensed phase at the saturation curve, i.e. at the vapor pressure p (the subscript 'sat' denotes a derivative along the saturation line; R is the molar gas constant).

The SimCor than starts from the Clapeyron equation in the form

$$\Delta H' = RT^{2} (\mathrm{d} \ln p / \mathrm{d}T)_{\mathrm{sat}} \tag{S3}$$

which relates the vapor pressure p to $\Delta_{\mathrm{cd}}^{\mathrm{g}} H_{\mathrm{m}}$ and the *pVT* behavior of the coexisting phases. The differentiation yields an equation relating vapor pressures to heat capacities

$$\Delta C' = R \left\{ \mathrm{d} \left[T^{2} \left(\mathrm{d} \ln p / \mathrm{d} T \right) \right] \Big/ \mathrm{d} T \right\}_{\mathrm{sat}}. \tag{S4}$$

It is apparent that quantities $\Delta H'$ and $\Delta C'$ can be calculated exclusively from vapor pressure equation (Eqs. (S3) and (S4)) or from thermal properties ($\Delta_{\mathrm{cd}}^{\mathrm{g}} H_{\mathrm{m}}$ and $\Delta_{\mathrm{cd}}^{\mathrm{g}} C_{p,\mathrm{m}}^{0}$) and appropriate *pVT* corrections (Eqs. (S1) and (S2)). This means that after selecting a suitable vapor pressure equation it is possible to correlate simultaneously experimental values of p_{sat}, $\Delta_{\mathrm{cd}}^{\mathrm{g}} H_{\mathrm{m}}$, and $\Delta_{\mathrm{cd}}^{\mathrm{g}} C_{p,\mathrm{m}}^{0}$ as a function of temperature, resulting in a set of vapor pressure equation parameters which are valid in a combined temperature range of all input experimental values.

While calorimetry is a source of $\Delta_{\mathrm{cd}}^{\mathrm{g}} H_{\mathrm{m}}$, and $C_{p,\mathrm{m}}^{\mathrm{cd}}$, the heat capacity of real gas is obtained using

$$C_{p,\mathrm{m}}^{\mathrm{g}} = C_{p,\mathrm{m}}^{\mathrm{g}0} - T \int_{0}^{p_{\mathrm{sat}}} \left(\frac{\partial^{2} V_{\mathrm{m}}^{\mathrm{g}}}{\partial T^{2}} \right) \mathrm{d}p \tag{S5}$$

where $C_{p,\mathrm{m}}^{\mathrm{g}0}$ is isobaric heat capacity of ideal gas, evaluated usually from spectroscopic (or *ab initio*) vibrational frequencies by means of statistical thermodynamics, and $V_{\mathrm{m}}^{\mathrm{g}}$ is molar volume of gas phase.

Generally, the *pVT* term in Eq. (S1) (i.e. term $\Delta_{\mathrm{cd}}^{\mathrm{g}} z$) represents correction around (3 to 7) percent to $\Delta_{\mathrm{cd}}^{\mathrm{g}} H_{\mathrm{m}}$, at the normal boiling point temperature T_{b}, while *pVT* terms in Eq. (S2) can amount up to 40 percent of $\Delta_{\mathrm{cd}}^{\mathrm{g}} C_{p,\mathrm{m}}$ value (see Fig. 1 on page 28 in Růžička and

Majer [4]). Since data for exact evaluation of *pVT* corrections are generally not available, the *pVT* correction is usually expressed by means of second virial coefficients (and molar volumes of liquid/solid phase). As experimental second virial coefficients are typically not available for temperatures well below the normal boiling temperature T_{nbp}, estimation methods must be used. This means that the uncertainty of *pVT* corrections is high and limits inclusion of thermal properties in the SimCor. Thus to avoid the distortion of SimCor by errors in *pVT* description, $\Delta_{\mathrm{cd}}^{\mathrm{g}}H_{\mathrm{m}}$ can be included in the SimCor at saturated pressures smaller than approximately 10 kPa and heat capacity difference $\Delta_{\mathrm{cd}}^{\mathrm{g}}C_{p,\mathrm{m}}$ at saturated pressures less than approximately 1 kPa; in case of hydrogen-bonding compounds as in this work, the upper pressure limit was set to 500 Pa only.

When the volume of gaseous phase is expressed as $V_{\mathrm{m}}^{\mathrm{g}} = \frac{RT}{p} + B$, Eq. (S1) can be written as

$$\Delta H' = \frac{\Delta_{\mathrm{cd}}^{\mathrm{g}}H_{\mathrm{m}}}{1 + \frac{p_{\mathrm{sat}}}{RT}\left(B - V_{\mathrm{m}}^{\mathrm{cd}}\right)} \quad \text{(S6)}$$

and Eq. (S4) can be converted (after neglecting the pressure dependence of $V_{\mathrm{m}}^{\mathrm{cd}}$) to the form [2]

$$\Delta C' = \Delta_{\mathrm{cd}}^{\mathrm{g}}C_{p,\mathrm{m}}^{0} - T\frac{\mathrm{d}^2 B}{\mathrm{d}T^2}p_{\mathrm{sat}} - 2T\frac{\mathrm{d}\left(B - V_{\mathrm{m}}^{\mathrm{cd}}\right)}{\mathrm{d}T}\left(\frac{\mathrm{d}p}{\mathrm{d}T}\right)_{\mathrm{sat}} - T\left(B - V_{\mathrm{m}}^{\mathrm{cd}}\right)\left(\frac{\mathrm{d}^2 p}{\mathrm{d}T^2}\right)_{\mathrm{sat}} \quad \text{(S7)}$$

The molar volume of saturated condensed phase $V_{\mathrm{m}}^{\mathrm{cd}}$ and its temperature derivative play negligible role at temperatures well below the normal boiling temperature, where Eqs. (S6) and (S7) are applied.

Several estimation methods for the second virial coefficient can be found in the literature. We prefer the method suggested by Tsonopoulos [5], as it provides also (empirical) corrections for polar compounds. Input parameters for this estimation method are given in Table S1. Note that in the temperature/pressure limits used for application of *pVT* correction even large change of input parameters would not significantly influence resulting vapor pressures and enthalpies of vaporization.

Table S6 Input Parameters Used for Evaluation of the Second Virial Coefficients by Tsonopoulos' Method [5]

Compound	T_c/ K	p_c/MPa	ω [a]	μ / D [b]
3H	582.4 [c]	3.058 [c]	0.540	1.60
2M2P	559.5 [c]	3.35 [d]	0.579	1.58
2M3P	576.0 [c]	3.46 [c]	0.508	1.55
3M2P	571.2 [d]	3.35 [d]	0.630	1.61
3M3P	576.6 [c]	3.52 [c]	0.451	1.55
22M1B	603.0 [d]	3.49 [d]	0.358	1.58
23M2B	555.6 [d]	3.47 [d]	0.566	1.60
33M2B	563.8 [d]	3.48 [d]	0.535	1.57

[a] acentric factor calculated by the SimCor.

[b] dipole moment calculated at the DFT B3LYP/6-311+G(2df,p) level of theory using R1TM approach [6] at 298.15 K.

[c] critical temperature and pressure recommended by Gude and Teja [7].

[d] critical temperature and pressure estimated by method of Nannoolal et al. [8] as implemented in Artist software package [9].

Any vapor pressure equation can be used in the SimCor method providing it is flexible enough to describe input properties within their assumed uncertainties. Different equations were tested by Růžička and Majer [3]. The Cox equation [10] (Eq. (3) in this paper) was used in most of our works as it is flexible and (in contrast to Wagner equation, which otherwise performs also quite well) does not require critical temperature and critical pressure.

The parameters of the vapor pressure equation are obtained by minimizing an objective function *S*, which is defined as

$$S=\sum_{i=1}^{m_p}\frac{\left(\ln p_{\mathrm{sat}}^{\mathrm{exp}}-\ln p_{\mathrm{sat}}^{\mathrm{calc}}\right)_i^2}{\sigma_i^2\ln p_{\mathrm{sat}}}+K_H^2\sum_{i=1}^{m_H}\frac{\left(\Delta H'^{\,\mathrm{exp}}-\Delta H'^{\,\mathrm{calc}}\right)_i^2}{\sigma_i^2\Delta H'}+K_C^2\sum_{i=1}^{m_C}\frac{\left(\Delta C'^{\,\mathrm{exp}}-\Delta C'^{\,\mathrm{calc}}\right)_i^2}{\sigma_i^2\Delta C'}. \quad \text{(S8)}$$

The quantities with the superscript "exp" relate to the experimental data ($\Delta H'^{\,\mathrm{exp}}$ are calculated by means of Eq. (S6) and $\Delta C'^{\,\mathrm{exp}}$ by means of Eq. (S7)). The quantities with the superscript "calc" are expressed from the Cox equation, Eq. (3), and Eqs. (S3) and (S4). The individual data points are weighted using the expected uncertainties of the experimental data. As a starting point, uncertainties claimed in the original data source are used. Very often, the original uncertainties are too optimistic and they are raised during SimCor or the whole dataset is even excluded from the correlation, when inconsistency with other types of data is observed. The quantity $\sigma_i^2\ln p_{\mathrm{sat}}$ is obtained from errors in temperature (σT) and pressure (σp)

$$\sigma_i^2\ln p_{\mathrm{sat}}=\left(\frac{\sigma p}{p}\right)^2+\left(\frac{\mathrm{d}\ln p}{\mathrm{d}T}\right)^2(\sigma T)^2. \quad \text{(S9)}$$

$\sigma_i^2\Delta C'$ is estimated from the expected uncertainties in heat capacities of respective phases

$$\sigma_i^2\Delta C'=\left(\sigma C_{p,\mathrm{m}}^{\mathrm{g}}\right)^2+\left(\sigma C_{p,\mathrm{m}}^{\mathrm{cd}}\right)^2 \quad \text{(S10)}$$

$\sigma C_{p,\mathrm{m}}^{\mathrm{cd}}$ for values included in SimCor (given in bold in Table 2) calculated from Eq. (1) with parameters from Table 3 was set to 1 percent for data from Tian-Calvet calorimeters. $\sigma C_{p,\mathrm{m}}^{\mathrm{g}}$ is tabulated set to 0.5 to 0.9 percent (see Table S5) for $C_{p,\mathrm{m}}^{\mathrm{g}}$. Weighing factors K_H^2 and K_C^2 are set to unity unless inconsistency between the different types of data is observed. Whenever possible, the inconsistency is resolved by new measurements instead of by changing K_H^2 and K_C^2.

Section S4. Recommended Vaporization Enthalpies

The enthalpy of vaporization can be calculated using the Cox equation, Eq. (3), with parameters given in Table 3 and *pVT* behavior using Eq. (S6). This requires the knowledge of the second virial coefficient *B*, which was estimated using the Tsonopoulos method [5]. For the reader's convenience, recommended values of $\Delta_l^g H_m$ are tabulated in Table S7 at discrete temperatures.

Table S7. Recommended Enthalpies of Vaporization of compouns of this work.

T / K	$\Delta_l^g H_m$ / kJ·mol^{-1}			
	3H [a]	2M2P [a]	2M3P [a]	3M2P [b]
260	62.42	58.81	59.60	60.97
265	61.94	58.38	59.10	60.49
270	61.44	57.92	58.57	59.99
275	60.91	57.43	58.02	59.47
280	60.35	56.92	57.44	58.93
285	59.78	56.38	56.83	58.38
290	59.18	55.82	56.20	57.81
295	58.56	55.24	55.55	57.22
300	57.93	54.64	54.87	56.62
305	57.27	54.01	54.18	56.00
310	56.60	53.36	53.47	55.37
315	55.92	52.69	52.74	54.73
320	55.21	52.00	52.00	54.07
325	54.50	51.29	51.25	53.40
330	53.77	50.57	50.49	52.72
335	53.03	49.83	49.71	52.02
340	52.29	49.07	48.93	51.32
345	51.53	48.29	48.15	50.61
350	50.76	47.50	47.36	49.89
355	49.99	46.70	46.57	49.16
360	49.21	45.88	45.78	48.42
365	48.43	45.06	44.99	47.68
370	47.65	44.23	44.21	46.94
375	46.87	43.39	43.44	46.19
380	46.09	42.54	42.67	45.44
385	45.31	41.70	41.92	44.69
390	44.54	40.85	41.18	43.94
395	43.77	40.01	40.46	43.19
400	43.01	39.17	39.76	42.45

T / K	$\Delta_l^g H_m$ / kJ·mol[-1]			
	3M3P [b]	22M1B [b]	23M2B [b]	33M2B [b]
260	58.30	60.46	56.97	56.32
265	57.82	60.05	56.52	55.84
270	57.31	59.63	56.03	55.34
275	56.77	59.18	55.52	54.82
280	56.19	58.71	54.97	54.28
285	55.59	58.22	54.40	53.72
290	54.95	57.71	53.80	53.15
295	54.29	57.18	53.17	52.56
300	53.61	56.64	52.53	51.96
305	52.90	56.08	51.86	51.34
310	52.18	55.51	51.17	50.72
315	51.44	54.92	50.47	50.08
320	50.68	54.32	49.75	49.44
325	49.91	53.71	49.01	48.79
330	49.13	53.09	48.27	48.14
335	48.34	52.47	47.51	47.49
340	47.54	51.83	46.75	46.83
345	46.74	51.18	45.98	46.18
350	45.95	50.53	45.21	45.53
355	45.15	49.88	44.44	44.88
360	44.36	49.22	43.67	44.24
365	43.57	48.56	42.90	43.60
370	42.79	47.89	42.14	42.98
375	42.03	47.23	41.39	42.36
380	41.29	46.57	40.66	41.76
385	40.56	45.91	39.93	41.17
390	39.85	45.26	39.23	40.60
395	39.17	44.62	38.55	40.04
400	38.52	43.98	37.89	39.50

[a] The uncertainties of tabulated $\Delta_l^g H_m$ reflect the uncertainties of the input data and are similar to those established for 1-alkanols in previous paper [6], i.e. they are less than 1 percent up to ca 360 K, and increase to 1.5 percent at 400 K due to uncertainty in *pVT* correction according to Eq. (S6).

[a] The uncertainties of tabulated $\Delta_l^g H_m$ reflect the uncertainties of the input data and are below one percent in the temperature range where vapor pressure are available (T<310 K), and increase to 2 percent at 400 K due to uncertainty in *pVT* correction according to Eq. (S7).

Section S5. Comparison of experimental and estimated vapor pressures

Estimation methods are not very successful when applied to vapor pressures of alkanols. Rarey and coworkers had to use additional term in their estimation method, yet the average relative deviation for alcohols in the "medium" pressure range (5.5 %) were significantly worse than for hydrocarbons (1.9 %). Updated method [11] improved average relative deviation to 4.1 %, thus confirming that estimation of vapor pressure of alcohols is challenging.
Figure S1 shows deviations of estimated vapor pressures (Rarey and coworkers [11], as implemented in the Artist software package [9]) from values recommended in this work as a function of temperature. The estimation method of Rarey and coworkers [11] is based on data stored in the Dortmund database; it requires the normal boiling point as an input. For this purpose, normal boiling point temperature was estimated using the method by Rarey and coworkers [12].

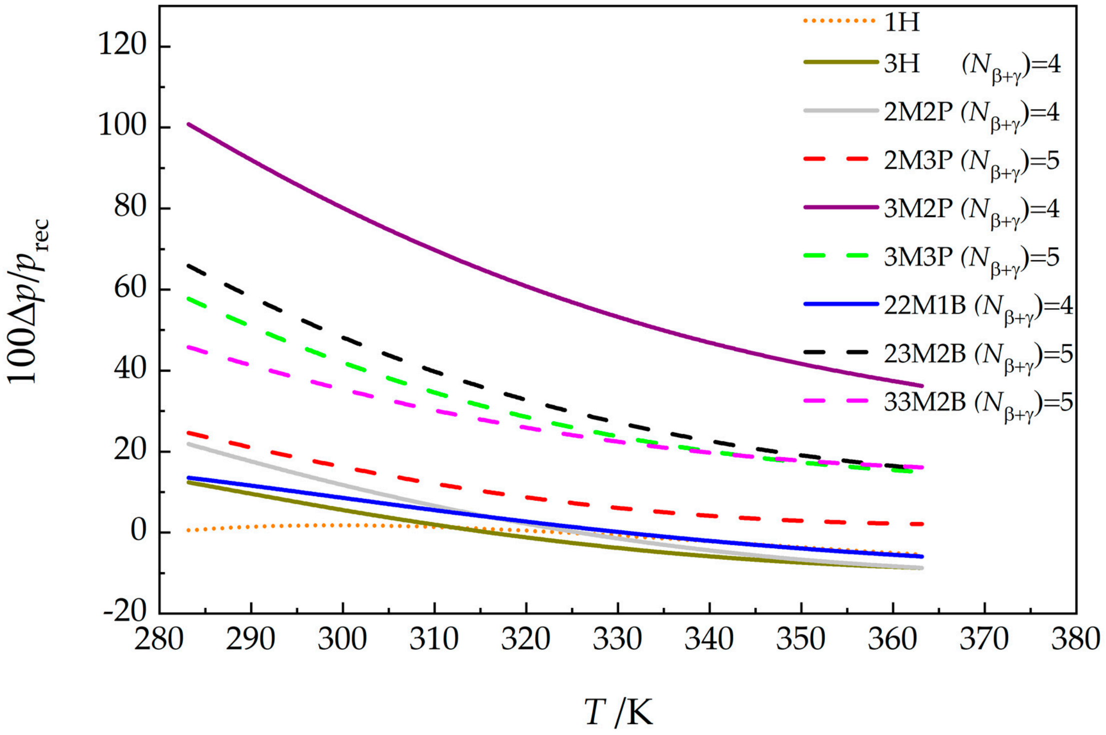

Figure S1. Relative deviations of estimated vapor pressures p_{est} by method of Rarey and coworkers [9,11,12] from values of this work p_{rec} (calculated from Cox equation, Eq. (3) with parameters from Table 3). Δp= p_{estr}- p_{rec}. Value for 1-heptanol (1H) is given for comparison; deviation of 1 H estimated vapor pressures is however substantially larger for vapor pressures lower and higher than those shown in this figure, see Figure S5 of our previous paper [6].

Section S6. Extrapolation of vapor pressures using GLC-ACRT methodology

This section contains Table S8 with values of relative activity coefficients at infinite dilution (γ_{rel}^{∞}) defined by Eq. (2), which are shown in Figure 8 and used to determine parameters *a* and *b* in Eq. (4) (Table S9), used for *p* extrapolation (Section 3.5).

Table S8. Relative activity coefficients γ_{rel}^{∞} defined by Eq. (2),

	3H	2M2P	2M3P	3M2P(1)	3M2P(2)	3M3P	22M1B	23M2B	33M2B
					Reference: 3H				
283.15		1.108	1.438	1.066	1.112	1.546	1.057	1.546	1.674
288.15		1.110	1.423	1.061	1.103	1.531	1.044	1.533	1.639
293.15		1.111	1.408	1.055	1.096	1.520	1.032	1.521	1.606
298.15		1.112	1.396	1.051	1.087	1.510	1.023	1.509	1.574
303.15		1.110	1.378	1.046	1.077	1.493	1.011		
308.15			1.364	1.038	1.070		1.006		
					Reference: 2M2P				
283.15	0.902		1.297	0.962	1.003	1.395	0.954	1.395	1.510
288.15	0.901		1.282	0.956	0.993	1.379	0.941	1.381	1.476
293.15	0.900		1.268	0.949	0.987	1.368	0.929	1.369	1.446
298.15	0.900		1.256	0.946	0.978	1.358	0.920	1.358	1.416
303.15	0.901		1.242	0.942	0.970	1.346	0.911		
					Reference: 2M3P				
283.15	0.696	0.771		0.742	0.774	1.075	0.735	1.075	1.164
288.15	0.703	0.780		0.746	0.775	1.076	0.734	1.077	1.152
293.15	0.710	0.789		0.749	0.779	1.079	0.733	1.080	1.140
298.15	0.716	0.796		0.753	0.779	1.082	0.732	1.081	1.128
303.15	0.726	0.805		0.759	0.782	1.084	0.734		
308.15	0.733			0.761	0.784		0.737		

Table S9. Parameters *a* and *b* of Eq. (4)

	Reference: 3H				Reference: 2M2P				Reference: 2M3P			
	a	*b*	n^a	R^2	*a*	*b*	*n*	R^2	*a*	*b*	*n*	R^2
3H	--	--	--	--	0.92041	$-6.67749 \cdot 10^{-5}$	5	0.297[b]	0.271158	$1.49767 \cdot 10^{-3}$	6	0.998
2M2P	1.08599	$8.22115 \cdot 10^{-5}$	5	0.296[b]	--	--		--	0.291149	$1.69592 \cdot 10^{-3}$	5	0.999
2M3P	2.26953	$-2.93730 \cdot 10^{-3}$	6	0.998	2.06954	$-2.73140 \cdot 10^{-3}$	5	0.998	--	--		
3M2Pa	1.37083	$-1.07549 \cdot 10^{-3}$	6	0.993	1.24126	$-9.89984 \cdot 10^{-4}$	5	0.980	0.512594	$8.08290 \cdot 10^{-4}$	6	0.993
3M2Pb	1.59368	$-1.70099 \cdot 10^{-3}$	6	0.997	1.46274	$-1.62495 \cdot 10^{-3}$	5	0.995	0.654825	$4.18546 \cdot 10^{-4}$	6	0.965
3M3P	2.25566	$-2.50979 \cdot 10^{-3}$	5	0.993	2.06219	$-2.36395 \cdot 10^{-3}$	5	0.994	0.944257	$4.60140 \cdot 10^{-4}$	5	0.977
22M1B	1.64801	$-2.09437 \cdot 10^{-3}$	6	0.990	1.55039	$-2.11291 \cdot 10^{-3}$	5	0.993	0.720375	$4.70349 \cdot 10^{-5}$	6	0.072[b]
23M2B	2.24521	$-2.47047 \cdot 10^{-3}$	4	0.999	2.09509	$-2.47540 \cdot 10^{-3}$	4	0.997	0.965532	$3.88443 \cdot 10^{-4}$	4	0.960
33M2B	3.55262	$-6.63852 \cdot 10^{-3}$	4	0.999	3.27758	$-6.24634 \cdot 10^{-3}$	4	0.998	1.849278	$-2.41959 \cdot 10^{-3}$	4	0.999

[a] Number of datapoints used for parameters determination.

[a] The coefficient of determination R^2 is very low since γ_{rel}^{∞} is very veak function of *T*.

References

1. Štejfa, V.; Fulem, M.; Růžička, K. First-principles calculation of ideal-gas thermodynamic properties of long-chain molecules by R1SM approach—Application to n-alkanes. *J. Chem. Phys.* **2019**, *150*, 224101, doi:https://doi.org/10.1063/1.5093767.
2. King, M.B.; Al-Najjar, H. Method for correlating and extending vapor pressure data to lower temperatures using thermal data. Vapor pressure equations for some *n*-alkanes at temperatures below the normal boiling point. *Chem. Eng. Sci.* **1974**, *29*, 1003-1011, doi:https://doi.org/10.1016/0009-2509(74)80092-8.
3. Růžička, K.; Majer, V. Simple and controlled extrapolation of vapor pressures toward the triple point. *AIChE J.* **1996**, *42*, 1723-1740, doi:https://doi.org/10.1002/aic.690420624.
4. Růžička, K.; Majer, V. Simultaneous treatment of vapor pressures and related thermal data between the triple and normal boiling temperatures for *n*-alkanes C_5-C_{20}. *J. Phys. Chem. Ref. Data* **1994**, *23*, 1-39, doi:https://doi.org/10.1063/1.555942.
5. Tsonopoulos, C. Empirical correlation of second virial coefficients. *AIChE J.* **1974**, *20*, 263-272, doi:https://doi.org/10.1002/aic.690200209.
6. Pokorný, V.; Štejfa, V.; Klajmon, M.; Fulem, M.; Růžička, K. Vapor Pressures and Thermophysical Properties of 1-Heptanol, 1-Octanol, 1-Nonanol, and 1-Decanol: Data Reconciliation and PC-SAFT Modeling. *J. Chem. Eng. Data* **2021**, *66*, 805-821, doi:https://doi.org/10.1021/acs.jced.0c00878.
7. Gude, M.; Teja, A.S. Vapor-Liquid Critical Properties of Elements and Compounds. 4. Aliphatic Alkanols. *J. Chem. Eng. Data* **1995**, *40*, 1025-1036, doi:https://doi.org/10.1021/je00021a001.
8. Nannoolal, Y.; Rarey, J.; Ramjugernath, D. Estimation of pure component properties: Part 2. Estimation of critical property data by group contribution. *Fluid Phase Equilib.* **2007**, *252*, 1-27, doi:https://doi.org/10.1016/j.fluid.2006.11.014.
9. *Artist software*; DDBST Software and Separation Technology GmbH: Oldenburg, Germany, 2019; Volume p. 2019.2010.0151
10. Cox, E.R. Hydrocarbon vapor pressures. *Ind, Eng. Chem.* **1936**, *28*, 613-616, doi:https://doi.org/10.1021/ie50317a029.
11. Moller, B.; Rarey, J.; Ramjugernath, D. Estimation of the vapour pressure of non-electrolyte organic compounds via group contributions and group interactions. *J. Mol. Liq.* **2008**, *143*, 52-63, doi:https://doi.org/10.1016/j.molliq.2008.04.020.
12. Nannoolal, Y.; Rarey, J.; Ramjugernath, D.; Cordes, W. Estimation of pure component properties: Part 1. Estimation of the normal boiling point of non-electrolyte organic compounds via group contributions and group interactions. *Fluid Phase Equilib.* **2004**, *226*, 45-63, doi:https://doi.org/10.1016/j.fluid.2004.09.001.
